# Describing the structural robustness landscape of bacterial small RNAs

**DOI:** 10.1186/1471-2148-12-52

**Published:** 2012-04-13

**Authors:** Guillermo Rodrigo, Mario A Fares

**Affiliations:** 1 Instituto de Biología Molecular y Celular de Plantas, Consejo Superior de Investigaciones Científicas, Universidad Politécnica de Valencia, Ingeniero Fausto Elio s/n, 46022 Valencia, Spain; 2 Department of Genetics, Smurfit Institute of Genetics, University of Dublin, Trinity College, Dublin, Ireland; 3 Institute of Systems and Synthetic Biology, Genopole, Université d' Évry Val d' Essonne, CNRS, 91034 Évry Paris, France

**Keywords:** Evolution, Evolvability, Plasticity, RNA structure, Robustness, Small RNA, Thermodynamics

## Abstract

**Background:**

The potential role of RNA molecules as gene expression regulators has led to a new perspective on the intracellular control and genome organization. Because secondary structures are crucial for their regulatory role, we sought to investigate their robustness to mutations and environmental changes.

**Results:**

Here, we dissected the structural robustness landscape of the small non-coding RNAs (sncRNAs) encoded in the genome of the bacterium *Escherichia coli*. We found that bacterial sncRNAs are not significantly robust to both mutational and environmental perturbations when compared against artificial, unbiased sequences. However, we found that, on average, bacterial sncRNAs tend to be significantly plastic, and that mutational and environmental robustness strongly correlate. We further found that, on average, epistasis in bacterial sncRNAs is significantly antagonistic, and positively correlates with plasticity. Moreover, the evolution of robustness is likely dependent upon the environmental stability of the cell, with more fluctuating environments leading to the emergence and fixation of more robust molecules. Mutational robustness also appears to be correlated with structural functionality and complexity.

**Conclusion:**

Our study provides a deep characterization of the structural robustness landscape of bacterial sncRNAs, suggesting that evolvability could be evolved as a consequence of selection for more plastic molecules. It also supports that environmental fluctuations could promote mutational robustness. As a result, plasticity emerges to link robustness, functionality and evolvability.

## Background

The discovery of the regulatory role of RNA has revolutionized our understanding of the molecular control and genome organization of living cells [1,2]. Small non-coding RNAs (sncRNAs) have been shown, both in prokaryotes and eukaryotes, to exert a tight control on gene expression. Of relevance, a particular secondary structure can confer a regulatory ability of translation [3], a catalytic activity [4], or an interfering ability to silence gene expression [5]. Importantly, a unique secondary structure is underlying all these mechanisms that, while preventing the degradation of the sncRNA, allows the interaction with and subsequent modification of other sncRNAs, mRNAs, or proteins. In summary, structures are fundamental in determining the potential roles of sncRNA and are, consequently, a fundamental component of the fitness of these molecules [6]. In an attempt to proof this point, many research groups have pursued identifying the footprints of natural selection on secondary structures of sncRNAs, although this remains elusive. In this study we test the hypothesis that selection indeed operates at this level, driving the evolution of sequences to codify structures that present beneficial traits for the organism.

Early studies tackled the structural robustness of RNA molecules [7,8], considering that robustness would be a beneficial trait. These approaches took advantage of a physicochemical model [9] that allows predicting the resulting phenotype (structure) from a given genotype (sequence). Recent computational studies have been mainly focused on precursors of miRNAs [10-13] and on viruses [14-16], and have suggested that natural RNA sequences are robust to mutations. However, as we show in this study, the statistical significance of the results depends on the choice of the reference sample of sequences. Moreover, whether robustness evolves driven directly by selection or is the by-product of the selection for another related magnitude remains highly controversial [17]. Despite their biological relevance, however, very little is known about the structural robustness of bacterial sncRNAs. Here, we propose a new definition of environmental robustness that better allows studying its relationship with mutational effects. In addition, we explore and describe the robustness landscape of bacterial sncRNAs and link it to functionality and evolvability.

Robustness to environmental perturbations is the cornerstone of biological adaptation and diversification. In bacteria, adaptation to environment requires of fundamental changes at the molecular level (i.e., mutations). These changes may lead to the functional divergence of proteins or RNAs that mediate the adaptation to the environment. Indeed, bacteria have the ability to rapidly accumulate beneficial mutations when growing in new environments [18]. If most of such functional mutations are destabilizing, owing to the fact they compromise ancestral functions, robustness to these mutations may fuel biological evolvability [19]. However, a strong robustness may buffer the accumulation of beneficial mutations. Hence, determining how robust are proteins or RNAs to environmental and genetic perturbations may unearth the rules of evolvability [20]. Our study reveals that plasticity evolved in natural sncRNAs, conferring evolvability to bacteria [8], and it also reveals that this magnitude modulates robustness.

## Results and discussion

### Robustness of small RNAs

Here, we define structural robustness as the sensitivity of an RNA molecule to perturbations: the greater the robustness of an RNA molecule, the more insensitive is to perturbations. To understand how RNA molecules respond to perturbations, we measured two types of robustness. First, environmental robustness (*R_e_*) accounts for the robustness to perturbations in the environment where the sncRNA lives. These perturbations come mostly from extra-cellular factors. We assumed that environmental perturbations alter the physicochemical properties for RNA folding. Under this assumption, we computationally induced environmental perturbations by altering the energetic parameters implemented in the thermodynamic model for the base-pairs interactions [9]. Alterations in the conformation of the sncRNA structure resulting from such perturbations were used to calculate *R_e_*. This assumption is justified because the thermodynamic model assumes a mathematical expression by decomposition, whose parameterization must be done against experimental data. Different sets of energetic parameters have been proposed [21], each of them being a relatively good approximation for making predictions. However, the model is certainly a simplification of the reality (effective model) and many more equations and parameters would be needed for a much more accurate calculation of free energies and RNA structures. Therefore, it is indebted to think that environmental conditions (e.g., concentration of ions) modulate the energetic parameters of this effective model, and that environmental robustness would be achieved by being insensitive to perturbations in those parameters [22]. Second, mutational robustness (*R_m_*) accounts for the robustness of structures to single point mutations in the sncRNA sequence. We provide formal mathematical definitions of these variables in section Methods. To perform the computation over RNA secondary structures we used Vienna RNA package [23].

**Figure 1:**
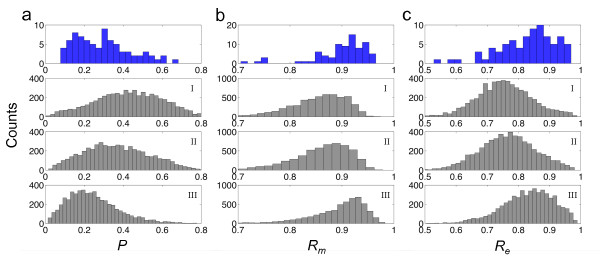
**Distributions of structural properties for sncRNAs**. Histogram of plasticity (*P*), mutational and environmental robustness (*R_m _*and *R_e_*) for the bacterial sncRNAs (blue bars) and for different samples (I, II and III) of artificially constructed sequences (gray bars). *U*-tests were applied to assess the statistical significance of the distributions. For *P*, the P-values of samples I, II and III are 8·10^-12^, 10^-4 ^and 0.0016, respectively. For *R_m_*, the P-values of the three samples are 7·10^-16^, 2·10^-12 ^and 0.38, respectively. For *R_e_*, the P-values of the three samples are 5·10^-13^, 3·10^-10 ^and 0.82, respectively.

We focused our study on the sncRNAs (79 genes) of the bacterium *Escherichia coli*, in particular the strain K12 MG1655 (Table S1) [24]. Among bacteria, *E. coli *is the one with more reported sncRNAs. And among the different strains of this bacterium, the K12 MG1655 is the one owning more sncRNAs. In bacteria, most of the riboregulation is based on antisense RNA-mediated repressions, although there are still few examples of activation. For instance, *dsrA *gene codifies for one sncRNA that represses the expression of *hns *gene (encoding for a Histone-like protein) in *E. coli *by inducing a loop in the mRNA, while it activates the expression of *rpoS *gene (encoding for a sigma factor for stress response regulation) that is under the control of a leader sequence able to sequester the ribosome binding site by forming a hairpin [25]. We calculated the mutational and environmental robustness for those bacterial sncRNAs. To do so, we first computed the thermodynamic ensembles of structures of all RNA molecules. We then applied several mutational and environmental perturbations to each of the sequences, recomputed the ensemble of structures of perturbed sequences, and calculated the base-pair distance between ensembles [26]. We finally averaged the results to compute *R_m _*and *R_e _*(Table S2, Figures 1, S1 and S2). In order to calculate *R_e _*we fixed the sequence and perturbed (several times) the energetic parameters, while in the case of *R_m _*we kept constant the energetic parameters and mutated (several times) the sequence.

**Figure 2:**
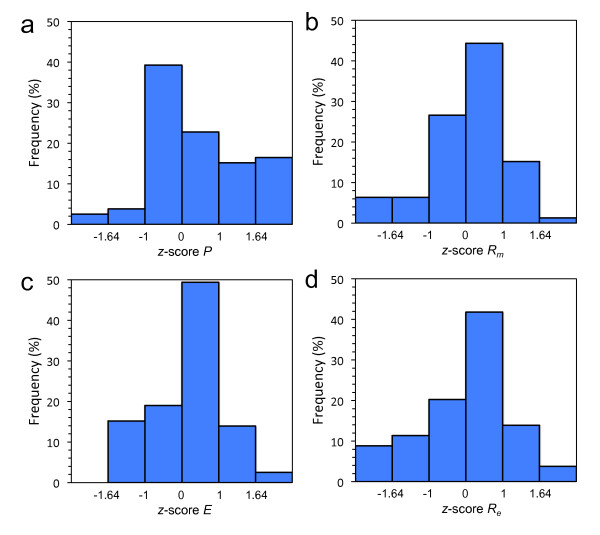
**Structural robustness landscape of the bacterial sncRNAs**. Histograms of *z*-scores for (a) plasticity (*P*), (b) mutational robustness (*R_m_*), (c) epistasis (*E*), and (d) environmental robustness (*R_e_*).

To assess the statistical significance of robustness values, we computed the *z*-scores associated to each sequence, with respect to the random population of structural analogs (Table S3). We constructed three different random samples of artificial sequences having the same minimal free energy (MFE) structures as the real sequences (see section Methods). We found that the statistical significance of the robustness (*z *> 1.64, P-value < 0.05), to both mutational and environmental perturbations, depends on the choice of the sample (Table S4). For instance, in sample I, 31.6% of the sequences was significantly robust to mutations, and 32.9% significantly robust to environmental perturbations. In addition, the entire set of sncRNAs was on average significantly robust (*U*-tests, P-values < 10^-10 ^for *R_m _*and *R_e_*) (Figure 1). These results are in agreement with previous studies [10,15], although caution should be taken in interpreting these values of robustness. In sample II, the fraction of significantly robust sncRNAs to mutations was reduced to 22.8%, while robustness to environment was reduced to 26.6% (Table S4). Despite these reductions, the results remain to be in stark agreement with a recent study [12]. In contrast to the two previous samples, sample III, the more unbiased one, allowed us to better identify the subset of significantly robust sncRNAs. In this sample, about 60% of genes were on average robust to both types of perturbations, mutational and environmental (*z *> 0), while only 1.3% (only 1 element) and 3.8% (only 3 elements) of genes were significantly robust to either mutational or environmental perturbations, respectively (Table S4). In addition, we did not find a significant enrichment in both types of robustness on average, comparing the whole set of bacterial sncRNAs against sample III (*U*-tests, P-values > 0.3 for *R_m _*and *R_e_*) (Figure 1). Figure 2 illustrates the structural robustness landscape of bacterial sncRNAs, using this last sample. Our results indicate that bacterial sncRNAs are not robust with respect to random sequences, and the comparative of the results for different null models indicate that previous analyses on the robustness of pre-miRNAs [10-12] should be revisited. To address this issue, we further applied our methodology to pre-miRNAs of *Caenorhabditis elegans*, and we found that they are not so robust as claimed before. More precisely, Szöllósi and Derényi reported for *C. elegans *37% of significantly robust pre-miRNAs, while we did not obtain any in the 100 sequences analyzed using an analog sample III.

### Robustness versus plasticity

**Figure 3:**
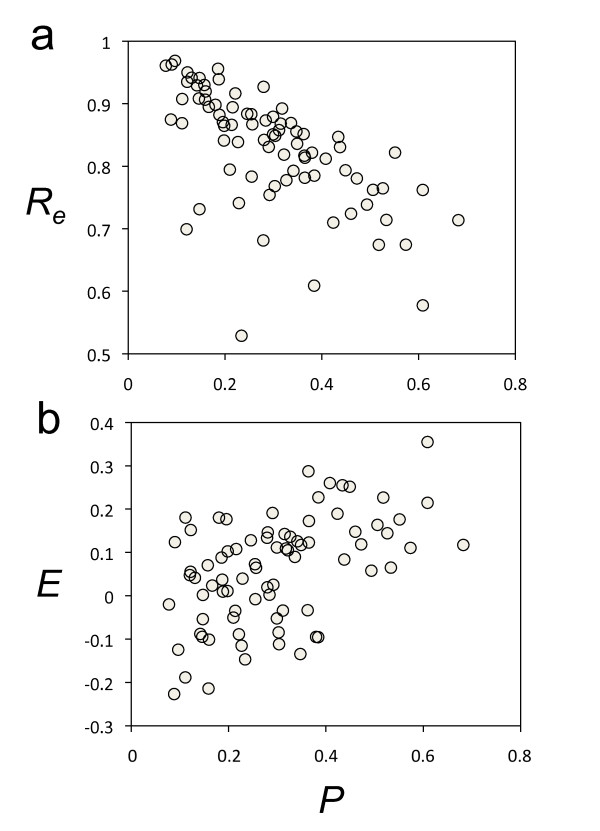
**Plasticity correlates with environmental robustness and epistasis**. (a) Scatter plot between plasticity (*P*) and environmental robustness (*R_e_*) for the bacterial sncRNAs. Spearman correlation coefficient = 0.396, P-value < 0.001. (b) Scatter plot between plasticity (*P*) and epistasis (*E*) for the bacterial sncRNAs. Spearman correlation coefficient = 0.728, P-value < 10^-6^.

In addition to the environmental robustness, we also took into account the plasticity (*P*) of the molecules (see section Methods). Sequences are more plastic when the thermodynamic ensemble of structures has higher variability [8]. In contrast to previous studies [11-13], here we disentangled plasticity (which relates to thermal stability) from environmental robustness. This division made it feasible distinguishing clear patterns of linked genetic and environmental robustness. We calculated the degree of plasticity of the sncRNAs and tested their significance as above (using sample III). We found that natural molecules were significantly more plastic, in terms of population, than artificial ones (*U*-test, P-value = 0.0016) (Figure 1). Specifically, 16.5% of the bacterial sncRNAs were significantly plastic (Figure 2), albeit the fraction of molecules significantly susceptible to the environment (*z *< -1.64) was much lower (about 8%). In addition, in terms of population, sncRNAs are not significantly robust to environmental perturbations, neither to mutational effects. As before, we also analyzed the plasticity for the pre-miRNAs of *C. elegans*, obtaining a significant enrichment of plasticity on average (*U*-test, P-value < 0.005), similar to bacterial sncRNAs. Not surprisingly we found a slight inverse correlation between *P *and *R_e _*(Figure 3a), because 1-*P *can also be understood as a kind of robustness to temperature (temperature as a particular environmental cue) [11]. Indeed, previous work pointed out that the higher the energy gap between the optimal and suboptimal structures of the thermodynamic ensemble (*P *close to 0), the higher is the robustness to mutations [27]. Of note, with a definition of environmental robustness of 1-*P*, our results would indicate that bacterial sncRNAs are on average more susceptible to environmental changes than artificial ones, while they are neither significantly susceptible nor robust to mutations.

The conclusion that bacterial sncRNAs are significantly plastic could entail important evolutionary and functional implications (Figure S3). First, plasticity could serve as a mechanism to diversify the functions of molecules, since a single genotype could yield multiple phenotypes (large thermodynamic ensemble of structures), even sncRNAs can adopt multistable states [28]. Second, plastic molecules have greater evolvability [8], which could lead to functional innovation (i.e., by increasing plasticity, the time of adaptation could be reduced). Third, the greater the plasticity, the larger structural changes can be after mutational or environmental perturbations (*t*-tests, P-values < 0.0001 for *R_m _*and *R_e_*, using the average of *P *to construct two subsets). Recently, it has been shown that robustness can correlate with evolvability but in a way strongly modulated by plasticity: intermediate robustness levels are optimal for fueling evolvability, where higher plasticity induces lower optimal robustness levels [20].

We also investigated epistasis (*E*) [29], the interaction of mutations, and its relationship with plasticity. In terms of population, we found that double mutations in bacterial sncRNAs tend to be antagonistic (*E *> 0) (*t*-test, P-value = 5·10^-5^). 70.9% of the sequences display *E *> 0, although with respect to sample III the statistical significance is reduced (*U*-test, P-value = 0.16). Antagonistic epistasis indicates that the effects of the first mutation at a nucleotide site provoke a disruption of the structure that is more severe than the one provoked by the effects of a second mutation at another site [29]. Accordingly, synergistic epistasis entails that single mutations will have a moderate impact on the structure. In fact, sncRNAs with synergistic epistasis displayed higher levels of mutational robustness (*t*-test, P-value < 0.0001, using *E *= 0 to construct two subsets). In principle, epistasis would tend to 0 when the two mutations fall down in the sequence with sufficient separation so that their effects are uncorrelated. Nucleotide sites that were detected to interact epistatically, both synergistically and antagonistically, were on average closer in the structure than expected by chance (Figure S4). In addition, we found a positive correlation between plasticity and epistasis (Figure 3b). Antagonistic epistasis thus comes from the fact that more plastic molecules are less robust. In this scenario, each individual is more sensitive to mutations (i.e., the deleterious mutants are quickly diluted while beneficial ones are fixed) and the population tends to accumulate beneficial genetic variability (Figure S3). Hence, our results are in tune with the suggestion that antagonistic epistasis would promote evolvability [30], and that evolvability and mutational robustness are inversely correlated, at least in the short term [31].

### Correlation between mutational and environmental robustness

**Figure 4:**
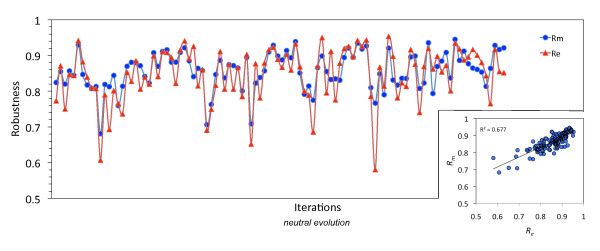
**Congruent evolution of mutational and environmental robustness**. Computation of mutational and environmental robustness (*R_m _*and *R_e_*) during a neutral evolution (acceptance of mutations that do not change the structure) of a MicA-like sncRNA (scatter plot in the inset). These two trajectories show a pattern of congruent evolution. One iteration corresponds to 100 mutations (order *O*(*L*)).

Based on our previous results, we sought to investigate whether mutational robustness correlates with our definition of environmental robustness, provided that the relationship between *R_m _*and 1-*P *has been already described [27]. We selected *micA *as a case study, although similar results could be derived with other sncRNAs, and we performed a neutral evolution process (accounting for potential compensatory mutations as described above to enlarge the sequence space). We found a significant correlation between the two types of robustness (Figure 4), although some punctual mutations can entail an opposite effect on these variables (Figure S5). By large, a sequence that evolves to increase its environmental robustness also increases its mutational robustness and *vice versa*. This reflects a clear dependency of these two magnitudes. Energetic parameters handle the structural robustness in both cases, but while environmental robustness is a global outcome, mutational robustness is local.

**Figure 5:**
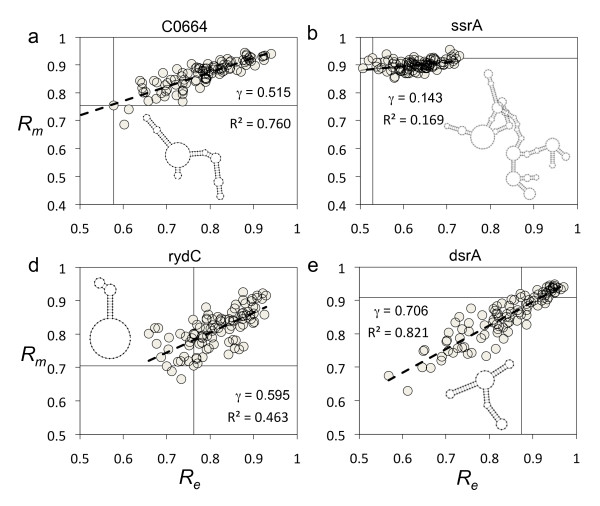
**Correlation between mutational and environmental robustness**. Computation of mutational and environmental robustness (*R_m _*and *R_e_*) for different sncRNAs and their corresponding structural analogs. The robustness of artificial sequences sharing the natural structure was evaluated (γ gives the value of the slope). Solid lines indicate the values for the natural sequence.

We further dissected and quantified robustness in the different sncRNA molecules. Interestingly, not all sncRNAs displayed the same level of robustness: a fraction of sncRNAs has not evolved to greater robustness (e.g., *C0664*), while the majority of them have reached suboptimal levels of robustness (e.g., *dsrA*) (Figures 5 and S6). The most robust gene was *micF*, to both mutations (*z *= 2.05) and environmental changes (*z *= 2.34), whereas the less robust gene was *C0064*, to both mutations (*z *= -5.87) and environmental changes (*z *= -5.82). MicF is a stress response sncRNA that targets the membrane protein OmpF and other genes related to chemotaxis [32,33]. However, its structure is very simple, with most of the nucleotides remaining unpaired, hence perturbations have minimal effects on the stability of this gene. On the contrary, C0064 is an enzyme with transferase activity that modifies rRNA and has been identified as the most plastic of the bacterial sncRNAs (*z *= 7.33). Environmental robustness strongly correlates with mutational robustness so that the promotion of one variable entails a proportional effect on the other (Figures 4, 5, and S7). Of relevance is the fact that the variability in sequence compositions that share a common structure was considerable and allowed unraveling a precise pattern by which the more robust molecules to environmental perturbations were also those more robust to mutational perturbations. This reinforces the fact that these two variables are not independent; hence the congruent evolution of the two robustness variables [11,12] in the case of bacterial sncRNAs becomes a plausible hypothesis.

Free-living bacteria are subjected to fluctuations in their environment. These perturbations affect the available resources that bacteria use for their development and reproduction, but also affect variables such as temperature, pressure, oxygen, metals, and concentration of ions. Changes in these variables may affect RNA folding, among other effects. It is then expected that molecules of free-living bacteria (which live in highly fluctuating environments) have evolved towards higher robustness to these environmental changes. It follows that environmental fluctuations may promote the evolution of mechanisms that confer robustness to such fluctuations. Afterwards, environmental robustness would provide sncRNAs with robustness to mutations, which is an inherent property of the molecule. In theory, direct selection for mutational robustness would only occur in organisms presenting high mutation rates such as viruses [34]. Thus, in populations with lower variability, mutational robustness could certainly be a side effect of selection for mechanisms that mitigate the effects of environmental perturbations [17]. In addition, the energetic features of the molecule manage its structural robustness to both mutational and environmental perturbations [22,27], and this explains the strong correlation between environmental and mutational robustness.

An illustrative test of this hypothesis would be the analysis of the robustness of sncRNAs of different bacteria, each subjected to different environmental fluctuations. Here we included in the analysis the endosymbiotic bacterium of aphid insects (*Buchnera aphidicola*), which lives in highly stable environments (i.e., devoid of fluctuations), among other free-living bacteria. However, these endosymbionts (also *Blochmannia floridanus*) have an extremely reduced genome [35] and hence very few or even none reported sncRNAs. Among all sncRNAs studied here, the gene codifying for one RNA component of the signal recognition particle, *ffs *[36], is highly conserved in many bacteria including *B. aphidicola*. Then we decided to focus our study on just this gene, observing that in *B. aphidicola *Ffs is significantly less robust than their Ffs homologs in other bacteria, which live in more fluctuating environments (Figure S8). Although these initial results do not constitute an exhaustive analysis to point out that evolution of robustness negatively correlates with environmental stability, they show that robustness can be compared among species and not only against artificial sncRNAs.

### Functionality of small RNAs

**Figure 6:**
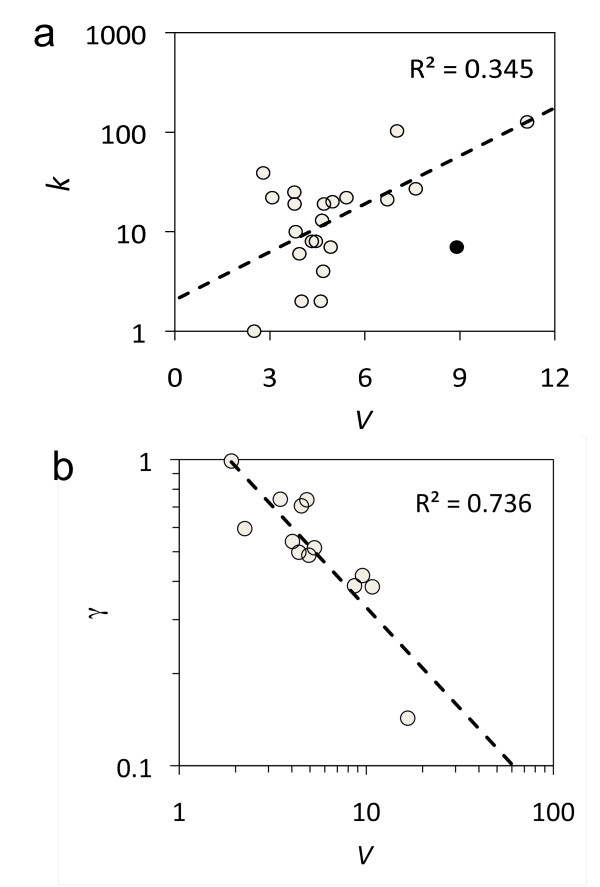
**Functionality of sncRNAs**. (a) Scatter plot between the connectivity degree of the sncRNAs (*k*) and degree of functionality (*V*). The outlier (black point) corresponds to gene *sgrS*, which is a particular sncRNA that also codifies for a small polypeptide (43 amino acids). (b) Scatter plot between degree of functionality (*V*) and *γ*, which is the slope of the linear regression between the mutational and environmental robustness for all sequences that have a common structure. For this plot, a representative subset of sncRNA structures was considered (genes *C0293, C0664, dsrA, ffs, gcvB, glmY, micA, oxyS, psrN, rydC, ryhB, sokC*, and *ssrA*).

To further dissect the robustness landscape, we calculated the degree of functionality (*V*) of the sncRNAs (see section Methods). The degree of functionality gives the total number of accessible regions in the sequence that may promote an interaction with another RNA molecule. Indeed, this degree would account simultaneously for complexity and functionality in sncRNA [37], with longer molecules presenting greater stability, more complex structures, and higher number of regions for potential interactions (Figure S9). The length (*L*) of the sncRNAs here studied goes from 53 to 436 nucleotides, but below 250 we find the majority of them (Figure S1). To show that the structural magnitude *V *is indeed a metric of functionality, we took the connectivity values (*k*) from a recent computational work that proposed an inferred network of Hfq-dependent sncRNAs [33]. We found a rough power-law relationship between *V *and *k *(Figure 6a). The higher the degree of functionality of an sncRNA, the more interactions can be established with mRNAs. Furthermore, the variance of the distribution of *R_m _*for several sequences sharing a common MFE structure depended on the functionality, while environmental robustness was insensitive to it (Figures 5 and 6b). This points out that more complex sncRNAs will display *per se *higher levels of mutational robustness (*t*-test, P-value < 0.0005, using the average of *V *to construct two subsets). Within a highly functional sequence, there are key nucleotides whose mutations provoke a significant disruption of the structure, whereas the majority of nucleotides have a more reduced impact on it. The sequence is hence on average robust to mutations. Similarly, studies relying on the topological properties of gene interaction networks have provided insights on why complex biological systems are more robust than simpler ones [38].

Of special interest are those molecules that are both plastic and environmentally robust. In principle, as we have shown, these two variables are negatively correlated. However, we observed that 17 bacterial sncRNAs presented this pattern. Among them, we highlight GcvB, IsrB, GlmZ, RseX, and RyhB. Interestingly, these sncRNAs present a high connectivity degree, especially GcvB. This could suggest that hub elements, in addition to increased degree of functionality, require high levels of plasticity to operate (*P *and *V *do not correlate, Figure S10). However, DicF and IsrA, which also establish many connections, do not exhibit this feature. Because the interaction network was inferred, these results should be interpreted with caution. Further studies are needed to address the important issue of linking robustness with functionality.

**Figure 7:**
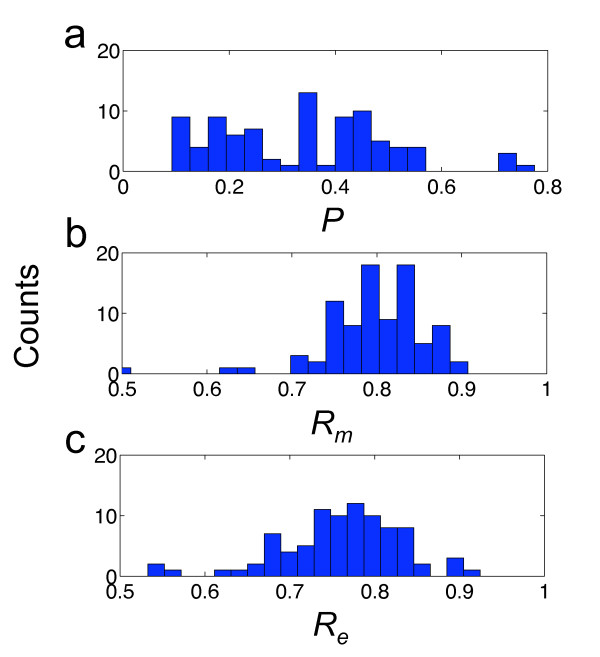
**Distributions of structural properties for tRNAs**. Histograms of plasticity (*P*), mutational and environmental robustness (*R_m _*and *R_e_*) for the bacterial tRNAs.

To further investigate the relationship between RNA function and robustness, we calculated *P*, *R_m _*and *R_e _*for the bacterial tRNAs (Figure 7) (sequences from GtRNAdb [39]). Because those tRNAs have a length between 74 and 93 nucleotides, we compared them against the sncRNAs with *L *< 100, although similar results were obtained for the whole set of sncRNAs. We observed that sncRNAs are significantly more robust, both to mutations and environmental perturbations, than tRNAs (*U*-test, P-value < 10^-8 ^for *R_m _*and *R_e_*), and significantly less plastic (*U*-test, P-value = 0.01, although the distributions are not normal). This could in principle indicate that the higher conservation of tRNAs is a consequence of low structural robustness, where one mutation would have a more severe effect than over a given sncRNA. This comparison points out differences in robustness of two RNA functional groups. However, the function of an RNA molecule is usually associated to the expression of one or various proteins. In particular, Hfq is an RNA chaperone that binds to sncRNAs for stabilization and assisting the interaction with the target mRNA [40]. We also know that tRNAs present great stability because they are recognized by endogenous enzymes that prevent degradation by nucleases, which allow tRNAs to accumulate in high concentrations within the cell [41]. Hence, it would be indebted to account for those endogenous enzymes to further link RNA robustness and functionality, for example by looking at mutations in the RNA sequence falling down in the protein recognition site.

### Limitations and further approaches

Of course, the use of the secondary structure as a fitness magnitude is a simplification. Future work could aim to determine the robustness to changes in gene expression by accounting for the interactions between sncRNAs and mRNAs [42], and also to assess the optimality of the natural riboregulators exploiting computational design methods [43]. Furthermore, the use of secondary structures to evaluate plasticity and robustness results in a limited framework. Certainly, a more accurate model, although at a high computational cost, would be the three-dimensional structure of the molecule, as we know that different types of interactions (not only Watson-Crick) exist [44]. In that way, packages such as iFoldRNA [45] could be exploited to carry out such robustness analyses.

Another important aspect corresponds to the uncertainty coming from transcription termination (a sncRNA also encodes a transcription terminator, usually the last hairpin of the structure is followed by a poly(U) tail). This process of transcription termination produces a population of sncRNAs with different lengths. The extra nucleotides in the transcript due to an inefficient termination (or the lack of nucleotides due to a premature termination) may influence the folding of the global structure [7]. Therefore, we could analyze the robustness of bacterial sncRNAs to this consequence, gaining accuracy with predictors of transcription termination [46].

In addition to compare natural RNAs against structural analogs, we could generate random sequences by adapting the nucleotide composition of the pool [47,48]. We could also use structural variables to complement sequence alignments in the detection of functional RNAs [48]. On the other hand, randomly generated sequences of sncRNAs could be a non-appropriate null model, because the evolution of natural sequences usually comes from shorter sequence distances [49]. To overcome this issue, we can analyze sncRNAs among different bacterial organisms. Although an sncRNA could not be significantly robust with respect to artificial sequences, it could be so with respect to analogs from other organisms (e.g., Ffs from *B. aphidicola *was significantly less robust than its analogs from free-living bacteria). This comparison indeed accounts for the short evolutionary distance and phylogenetic dependence.

## Conclusions

In this work, we used a computational approach to dissect the structural robustness landscape of the sncRNAs encoded in the genome of the bacterium *E. coli*. We identified that natural sncRNAs are not significantly robust to both mutational and environmental perturbations when compared against artificial, unbiased sequences. We also showed the dependence of the robustness analyses on different sets of artificial sequences. However, using the appropriate null model, we found a significant enrichment of plasticity in natural sequences. In contrast, previous studies claimed for significant robustness of natural pre-miRNAs [10-12], but this could reflect a caveat of the reference set of artificial sequences. By further applying our methodology to pre-miRNAs of *C. elegans*, we found that they are not so robust as claimed before but are significantly plastic. This is in tune with the results here presented for bacterial sncRNAs. Indeed, both bacterial sncRNAs and nematode pre-miRNAs appear as significantly more plastic on average, a trait that could promote evolvability [8]. This enhances the idea that evolvability, or the ability of finding beneficial or innovative mutations, could be a selected trait in bacterial sncRNAs.

In addition, our results can strengthen the understanding of the evolution of robustness and plasticity, concepts that have fueled enormous interest in the latest literature owing to their direct link with the promotion of adaptive evolution [20]. On the one hand, more functional (complex) structures would permit a larger number of RNA-RNA interactions and we have shown these structures display higher robustness levels. On the other hand, plasticity would promote evolvability and we have shown it is negatively correlated with robustness. The observation that plasticity positively correlates with epistasis (on average, significantly antagonistic in bacterial sncRNAs) supports the positive relationship between plasticity and evolvability, since antagonistic epistasis would promote evolvability [30]. This reflects a given modulator effect of plasticity on both robustness and evolvability. All in all, our study provides a quantitative, deep characterization of the complex map linking robustness, functionality and evolvability in bacterial sncRNAs.

## Methods

For a given sncRNA sequence (of length *L*), there is a thermodynamic ensemble (Ω) that contains the different suboptimal structures, each with a given free energy (*G_i_*) [50]. Thus, the partition function reads $Z=\underset{i\in \Omega }{\sum }\exp\left(-G_{i}/kT\right)$, and the free energy of the ensemble is *G *= -*kT *ln(*Z*). Then, the probability that the sncRNA folds into the structure *i *is given by $\Pi_{i}=\frac{\exp\left(-G_{i}/kT\right)}{Z}$. We assumed *T *= 37°C, then *kT *= 0.616 Kcal/mol. In this work, instead of comparing the MFE structures to analyze two different sequences, we compared the two ensembles of structures corresponding to the sequences. We introduced the base-pair distance between two structures (*d_BP_*), which is more accurate than the Hamming distance, to evaluate the difference between two structures [26]. The base-pair distance (*d_BP_*) between the different structures of Ω (*S_i _*denotes structure *i*), referred as intrinsic distance, is given by $d_{0}=\underset{i\in \Omega }{\sum }\underset{j\in \Omega }{\sum }d_{BP}\left(S_{i},S_{j}\right)\Pi_{i}\Pi_{j}$ (doubly probabilistically averaged). This magnitude accounts for the structural variability within Ω of a given sequence, and then allows defining plasticity (*P*). Lower values of *d_0 _*indicate that Ω is dominated by the MFE structure, while higher values correspond to ensembles with more suboptimal structures within a given energy gap. More plastic is a sequence when it presents more structural fluctuations at the equilibrium. Therefore, we defined plasticity as $P=\frac{2d_{0}}{L}$. This magnitude can then be used to distinguish very stable RNAs.

To compute *R_m _*we need to compare different mutant sequences. The average distance between structural ensembles after one single point mutation (*d_1_*) follows $d_{1}=\underset{i\in \Omega }{\sum }\underset{j\in \Omega_{1}}{\sum }d_{BP}\left(S_{i},S_{j}\right)\Pi_{i}\Pi_{j}-d_{0}$ (where Ω_1 _is the ensemble of mutants and Π*_j _*is calculated using the partition function of Ω_1_, denoted by *Z_1_*). Since *d_1 _*only accounts for one mutant, we need to average several calculations. Here 〈•〉 indicates average for perturbations and Δ• standard deviation. Hence, 〈*d*_1_〉 is the average structural distance after 1 single point mutation (*L *calculations). Then, we defined mutational robustness as $R_{m}=1-\frac{2\left\langle d_{1}\right\rangle }{L}$. As in the definition of *P*, we rescaled by *L*/2 to have an absolute value, since 〈*d_i_*〉 scales with *L *and because the number of base-pairs of a structure is bounded by *L*/2 (i.e., *d_BP _*between certain structure and the unfolded state is bounded by *L*/2). Certainly, the lower the distance, closer to 1 (maximum) should be the robustness. Here we considered that robustness follows a linear trend with the relative structural distance, although quadratic expressions could also be employed [13]. Analogously, we calculated the distance between structural ensembles after 2 single point mutations (*d_2_*), and the distance between ensembles after one environmental perturbation (*d_e_*), which was simulated as a random variation over the value of all the energetic parameters that define the model for base-pair interactions. For that, all parameters determining the energies for base pairing and stacking are perturbed simultaneously [22]. More in detail, to perform environmental perturbations, we took variations up to 20% of the nominal values following normal random distributions, i.e., being *β_0 _*the nominal value of an energetic parameter, the perturbed value reads *β *= (1+0.2*ξ*)*β*_0_, where *ξ *~ Ν(0,1). Hence, 〈*d*_2_〉 is the average structural distance after 2 single point mutations (10 *L *calculations), and 〈*d_e_*〉 is the average structural distance after an environmental perturbation (1,000 calculations). Then, we defined environmental robustness as $R_{e}=1-\frac{2\left\langle d_{e}\right\rangle }{L}$. We further defined epistasis as $E=1-\frac{\left\langle d_{2}\right\rangle }{2\left\langle d_{1}\right\rangle }$, which measures the interference between mutations. *E *> 0 means antagonistic epistasis (i.e., 〈*d*_2_〉 < 2〈*d*_1_〉, resulting in compensatory effects), while *E *< 0 synergistic epistasis (i.e., 〈*d*_2_〉 > 2〈*d*_1_〉, resulting in enhancement effects) [15].

In addition, for each sncRNA we computed its degree of functionality (*V*), given by $V=\underset{i\in \Omega }{\sum }V_{i}\Pi_{i}$ (probabilistically averaged), where *V_i _*is the number of times that a motif involving consecutively three free nucleotides and three bound nucleotides (in the 5' sense or in the 3') appears in the structure *i *of the ensemble. Two overlapping motifs were counted as a single event. While *V_i _*is a magnitude that corresponds to one structure, *V *corresponds to a sequence. We call this magnitude functionality because it quantifies the number of different mechanisms for potential interactions with further RNA molecules [2,42]. In other words, the degree of functionality accounts for the number of regions that may provide accessibility for RNA-RNA interactions. Moreover, *V_i _*is roughly proportional to the number of hairpins of the structure, and that metric of functionality also accounts for the complexity of the molecule.

Structural robustness was tested for significance by comparing it to a distribution of robustness values generated from a large set of artificially originated sequences. Artificial sequences shared the property of yielding the same MFE structures as the real sequences. For each sncRNA, we generated 69 random sequences, resulting in a population of 5,451 elements. Results were primary maintained when using smaller random populations. We constructed three different random samples. Sample I was obtained by iteratively solving the corresponding inverse folding problems using different initial sequences [10] with Vienna RNA package (default energetic parameters, dangles = 2, MFE objective) [23,51]. Notably, this allows sharing the MFE structure, but the thermodynamic ensembles may differ. Subsequently, sample II was obtained by combining inverse folding and neutral evolution, introducing mutations that do not change the MFE structure [12], thereby minimizing the bias introduced by the optimization method itself. For that, we performed *L *mutations. This process, nevertheless still produces biased sequences because mutations would be accumulated in regions with unpaired nucleotides (e.g., loops or tails). By definition, mutations affecting paired nucleotides are not neutral, with the exception of G-U/G-C paired regions. To counterbalance this bias, we constructed a sample III by which sequences were subjected to a neutral evolution process accounting for potential compensatory mutations (also *L *mutations). This process was based, in the case of paired nucleotides, on mutating the complementary nucleotides as well. Following this procedure, the simulated neutral evolution process accounts for both neutral single-point mutations and neutral base-pair mutations. This allowed enlarging considerably the sequence space and avoid more efficiently the bias produced by inverse folding methods.

## Competing interests

The authors declare no conflict, financial or non-financial, of interest. The authors declare that are not in contact with any organization that may gain or lose financially from the publication of this manuscript. The authors declare that are not applying for any patent relating to the content of this manuscript.

## Authors' contributions

GR conceived the work. GR performed the computational analyses. GR and MAF analyzed the data. GR and MAF wrote the manuscript.

## Supplementary Material

Additional file 1 **Figure S1 Histograms of the structural properties for the bacterial sncRNAs**. Click here for file

Additional file 2 **Figure S2 Plasticity modulates variability in robustness**. Scatter plots between the intrinsic distance (*d_0_*) and the standard deviations of the distances between structures after one (*Δd_1_*) or two mutations (*Δd_2_*) or environmental changes (*Δd_e_*) for the bacterial sncRNAs. Click here for file

Additional file 3 **Figure S3 Dependence of evolvability on structural properties**. Relationship scheme between plasticity (*P*), epistasis (*E*), mutational robustness (*R_m_*), and evolvability for bacterial sncRNAs. Click here for file

Additional file 4 **Figure S4**. Average effect of the location (relative distance) of mutations on epistasis using a large set of artificial sncRNAs. Click here for file

Additional file 5 **Figure S5 Robustness and neutral evolution**. Computation of mutational and environmental robustness (*R_m _*and *R_e_*) during a neutral evolution (acceptance of mutations that do not change the structure) of a MicA-like sncRNA. One iteration corresponds to one mutation. Click here for file

Additional file 6 **Figure S6 Mutational versus environmental robustness**. Scatter plot between mutational (*R_m_*) and environmental (*R_e_*) robustness for the bacterial sncRNAs, showing the gene name of the three frontier elements (genes *rydC*, *C0664 *and *ssrA*). Click here for file

Additional file 7 **Figure S7 Correlation between mutational and environmental robustness**. Scatter plot between the *z*-scores for environmental and mutational robustness (*R_e _*and *R_m_*), relative to sample III. Click here for file

Additional file 8 **Figure S8 Effect of environmental stability on robustness**. (a) Mutational and (b) environmental robustness (*R_m _*and *R_e_*) of gene *ffs *for different bacteria (*Buchnera aphidicola*, *Mycoplasma genitalium*, *Vibrio fischeri*, *Escherichia coli*, *Salmonella enterica*, *Citrobacter koseri*, *Serratia proteamaculans*, and *Pseudomonas putida*). * denotes statistical significance in a one-tailed *z*-test with (a) P-value = 0.059 and (b) P-value = 0.041. When including into the analysis 15 more strains of *E. coli *with different *ffs *sequences, we obtained (a) P-value = 0.005 and (b) P-value = 0.017. Click here for file

Additional file 9 **Figure S9 Length correlates with stability and functionality**. Scatter plots between length (*L*) and free energy of the ensemble (*G*) and degree of functionality (*V*) for the bacterial sncRNAs (*G *in Kcal/mol). Click here for file

Additional file 10 **Figure S10 Plasticity does not correlate with functionality**. Scatter plot between degree of functionality (*V*) and plasticity (*P*) for the bacterial sncRNAs. Click here for file

Additional file 11 **Table S1 Sequences for the small non-coding RNAs (sncRNAs) obtained from the genome of the bacterium *Escherichia coli *K12 MG1655**. Click here for file

Additional file 12 **Table S2 Structural properties for the bacterial sncRNAs**. These are length (*L*), free energy of the thermodynamic ensemble (*G*), degree of functionality (*V*), plasticity (*P*), mutational robustness (*R_m_*), epistasis (*E*), and environmental robustness (*R_e_*). Click here for file

Additional file 13 **Table S3 Statistical significance analysis results**. *Z*-scores for plasticity (*P*), mutational robustness (*R_m_*), environmental robustness (*R_e_*), and epistasis (*E*), for each sncRNA and relative to sample III. Click here for file

Additional file 14 **Table S4 Effect of random sample of sequences on robustness**. Sample I accounts for sequences obtained from inverse folding routines. Sequences of sample II were subsequently randomized by introducing neutral mutations (which not change the structure). Sequences of sample III were randomized by introducing neutral mutations and neutral pairs of mutations and compensatory mutations (for nucleotides in a stem). Using *z*-scores, we show the percentage of sncRNAs that are robust (*z *> 0) and significantly robust (*z_c _*= 1.64, P-value = 0.05). Click here for file
