# Supplementary figures and images for: Describing the structural robustness landscape of bacterial small RNAs
Source: BMC Evol Biol. 2012 Apr 13;12:52. doi: 10.1186/1471-2148-12-52 (PMC3368786; doi:10.1186/1471-2148-12-52)

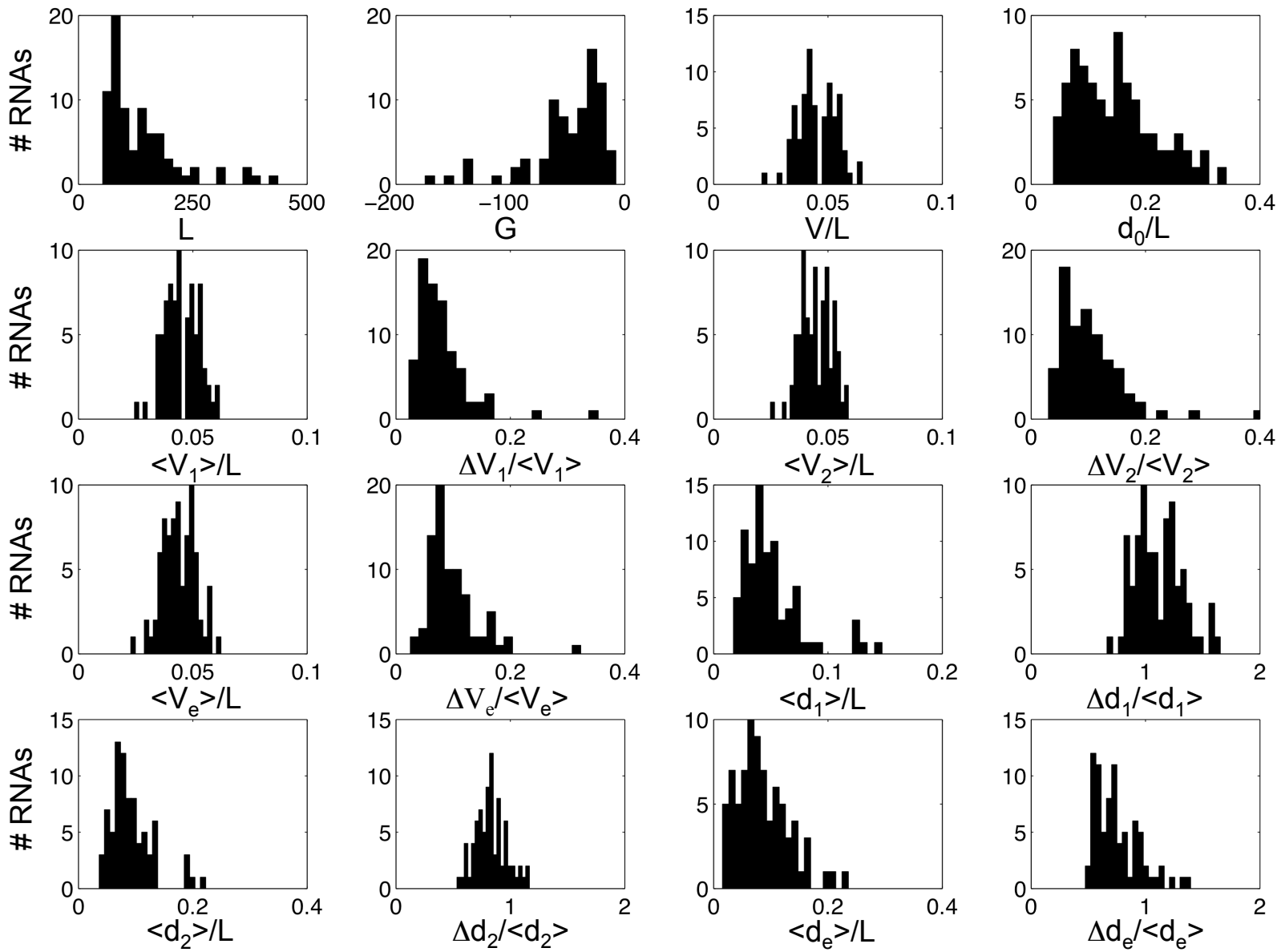

Supplement: Additional file 1 — Figure S1 Histograms of the structural properties for the bacterial sncRNAs. [file 1471-2148-12-52-S1.PDF]

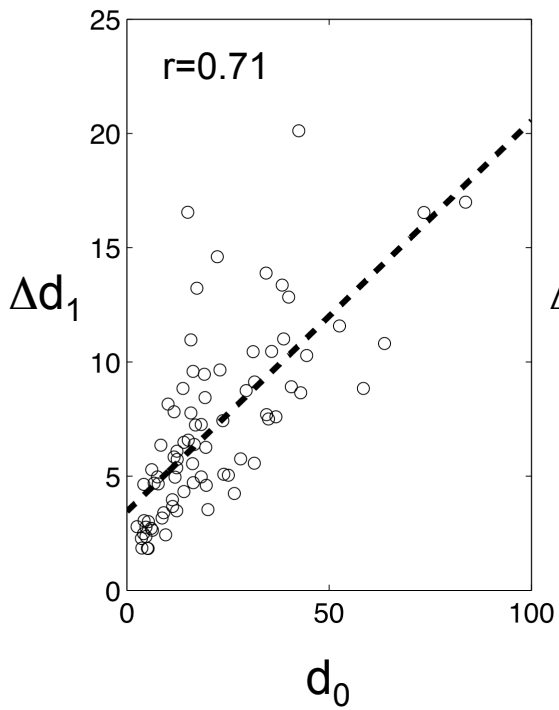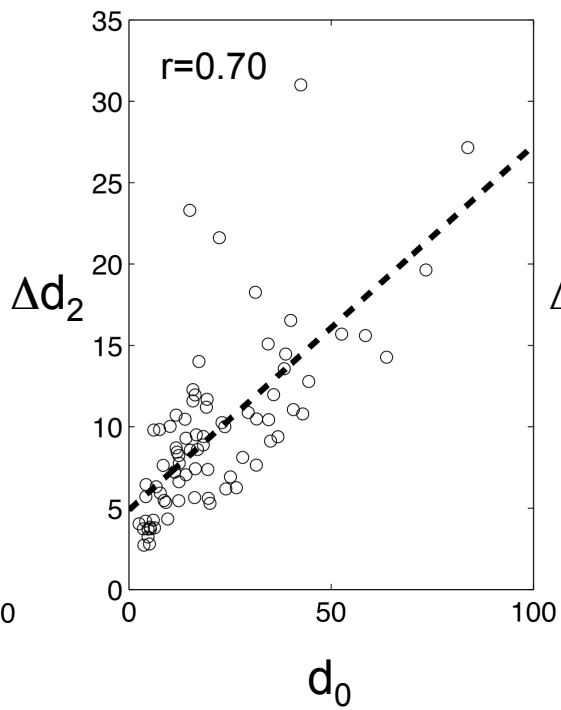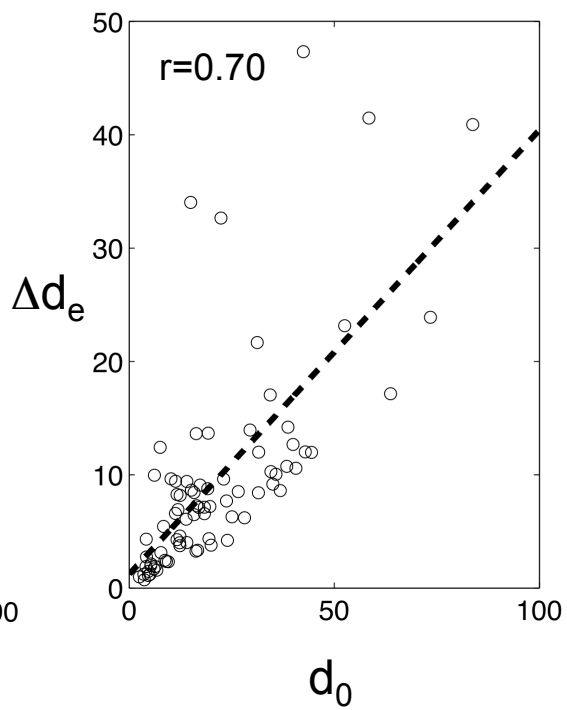

Supplement: Additional file 2 — Figure S2 Plasticity modulates variability in robustness. Scatter plots between the intrinsic distance (d0) and the standard deviations of the distances between structures after one (Δd1) or two mutations (Δd2) or environmental changes (Δde) for the bacterial sncRNAs. [file 1471-2148-12-52-S2.PDF]

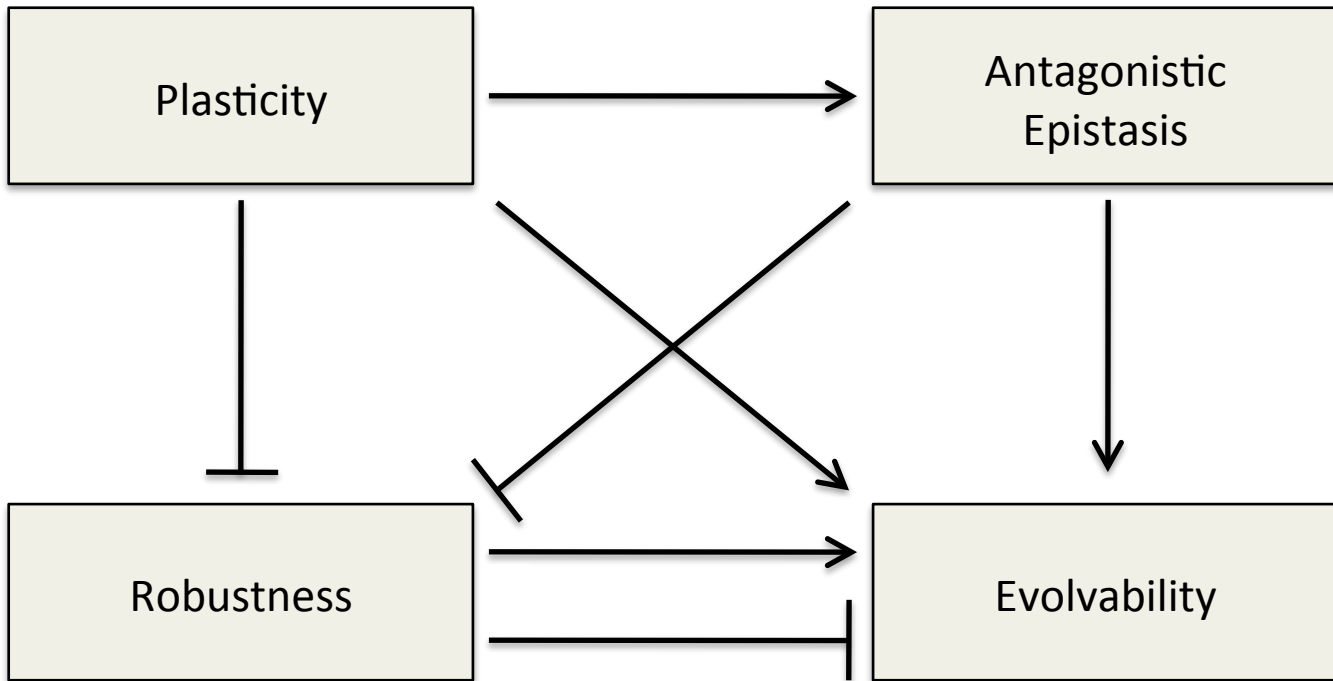

Supplement: Additional file 3 — Figure S3 Dependence of evolvability on structural properties. Relationship scheme between plasticity (P), epistasis (E), mutational robustness (Rm), and evolvability for bacterial sncRNAs. [file 1471-2148-12-52-S3.PDF]

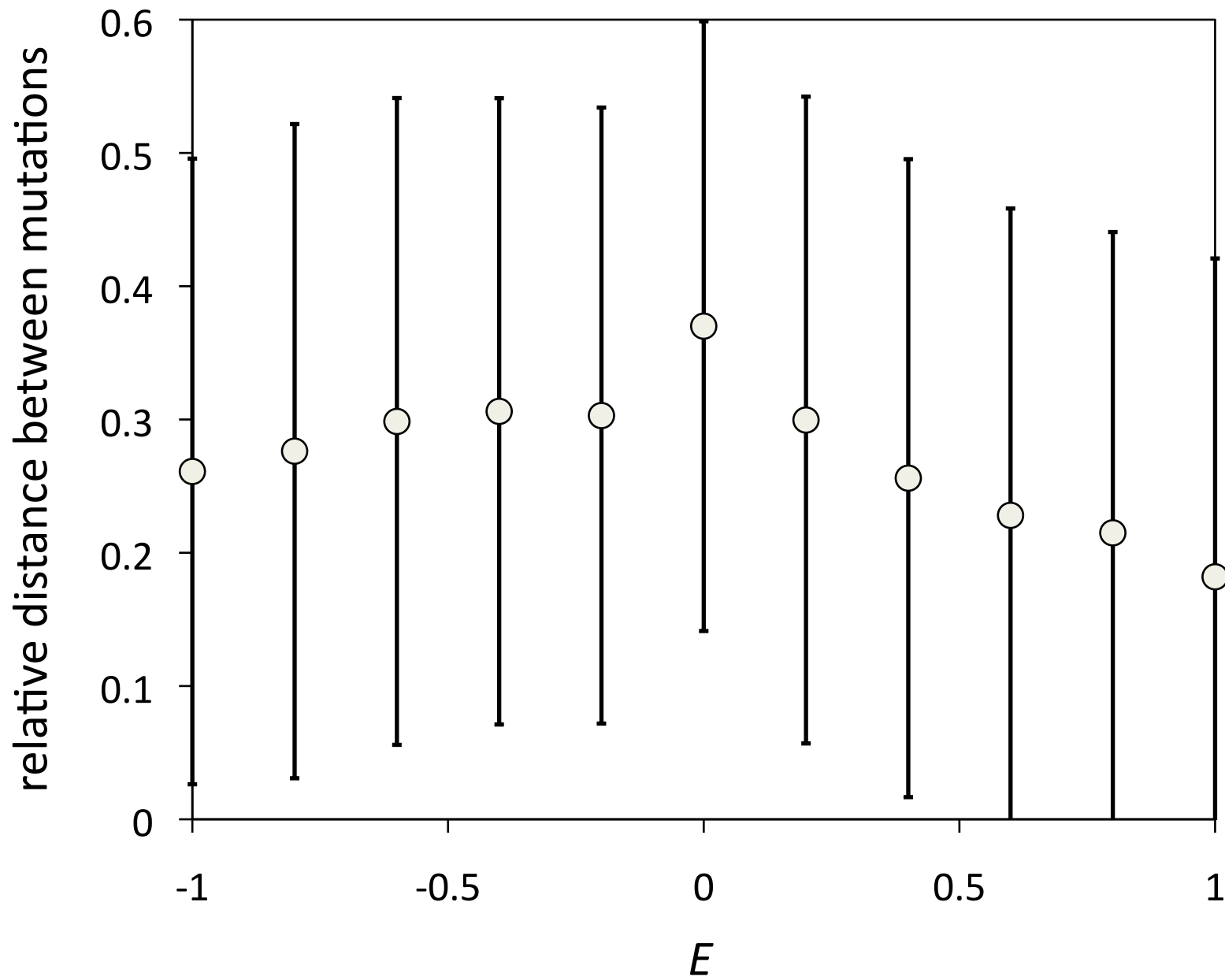

Supplement: Additional file 4 — Figure S4. Average effect of the location (relative distance) of mutations on epistasis using a large set of artificial sncRNAs. [file 1471-2148-12-52-S4.PDF]

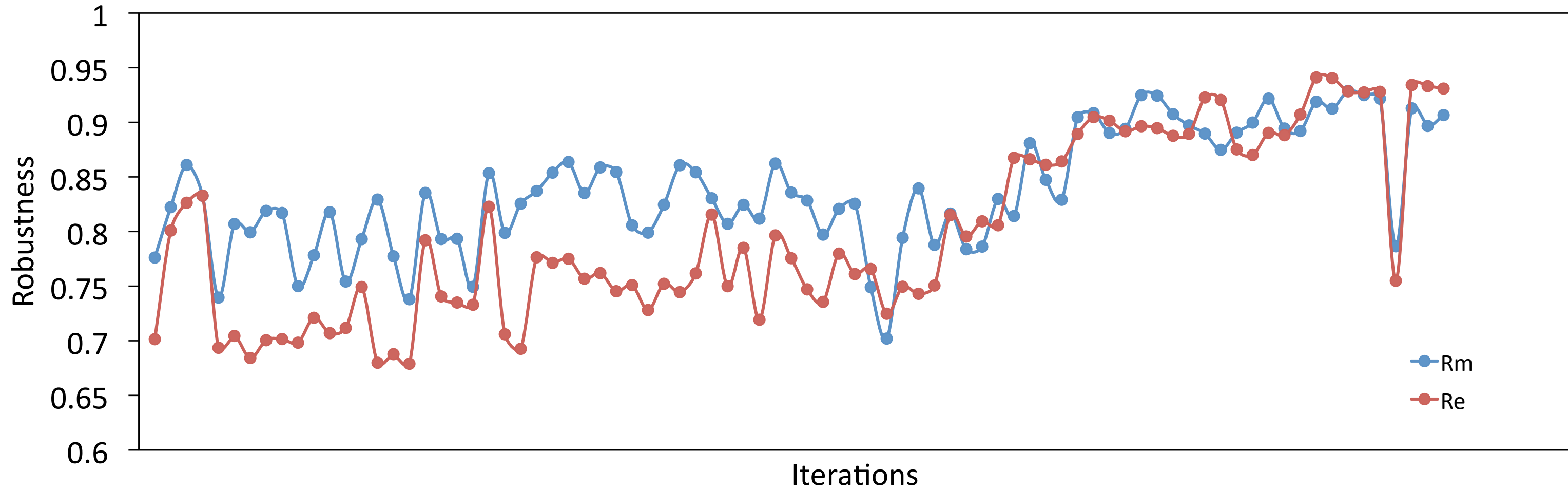

Supplement: Additional file 5 — Figure S5 Robustness and neutral evolution. Computation of mutational and environmental robustness (Rm and Re) during a neutral evolution (acceptance of mutations that do not change the structure) of a MicA-like sncRNA. One iteration corresponds to one mutation. [file 1471-2148-12-52-S5.PDF]

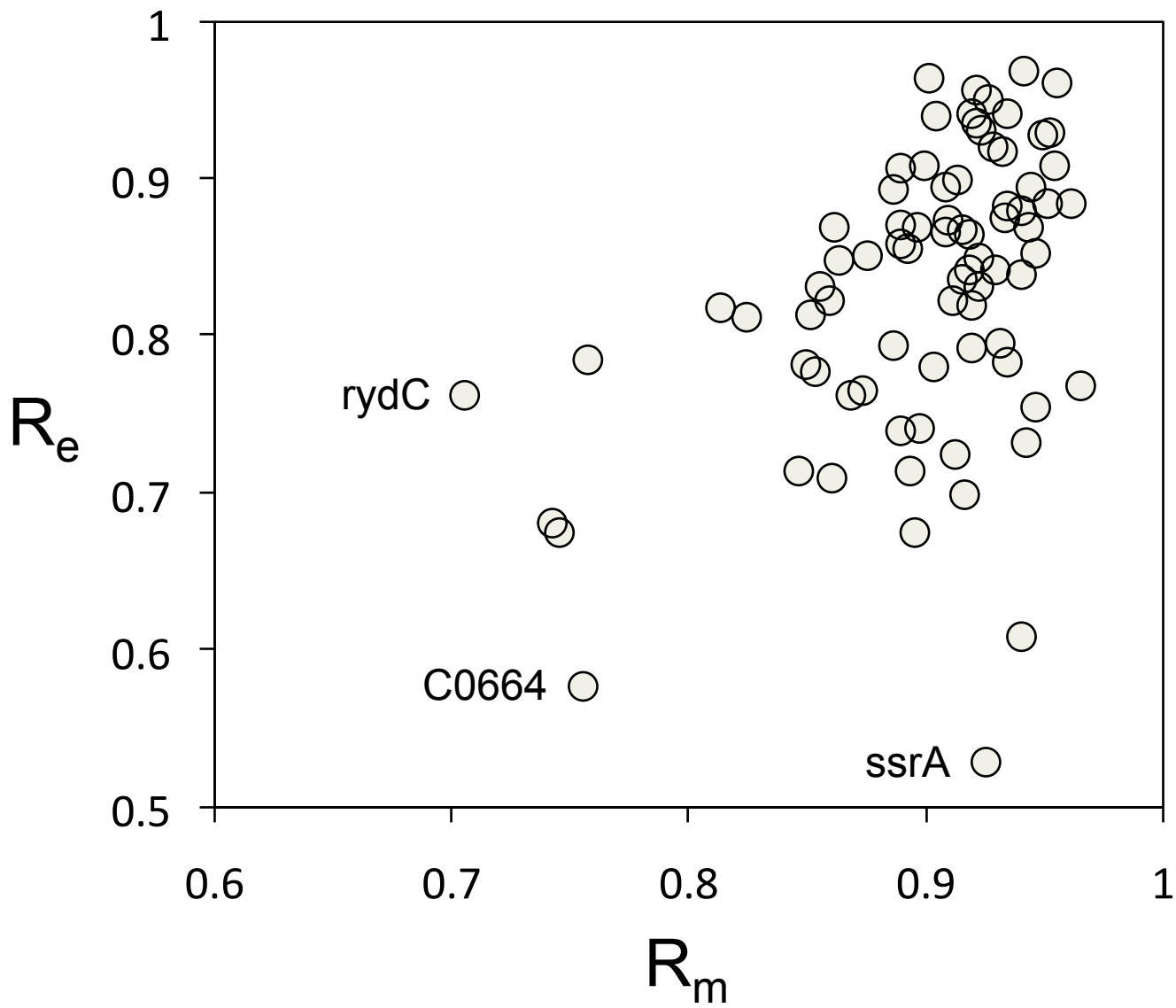

Supplement: Additional file 6 — Figure S6 Mutational versus environmental robustness. Scatter plot between mutational (Rm) and environmental (Re) robustness for the bacterial sncRNAs, showing the gene name of the three frontier elements (genes rydC, C0664 and ssrA). [file 1471-2148-12-52-S6.PDF]

z-score  $R_m$

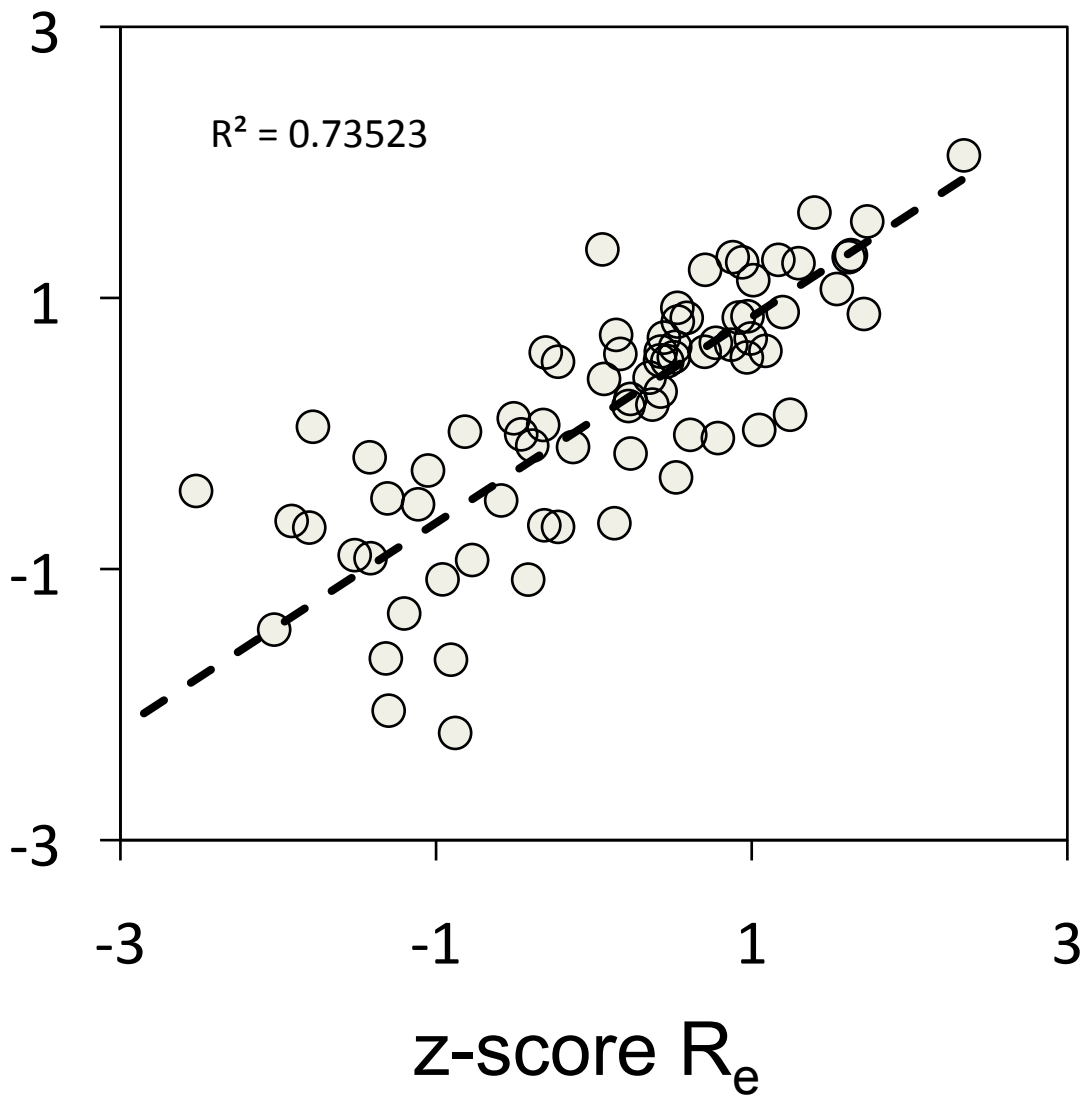

Supplement: Additional file 7 — Figure S7 Correlation between mutational and environmental robustness. Scatter plot between the z-scores for environmental and mutational robustness (Re and Rm), relative to sample III. [file 1471-2148-12-52-S7.PDF]

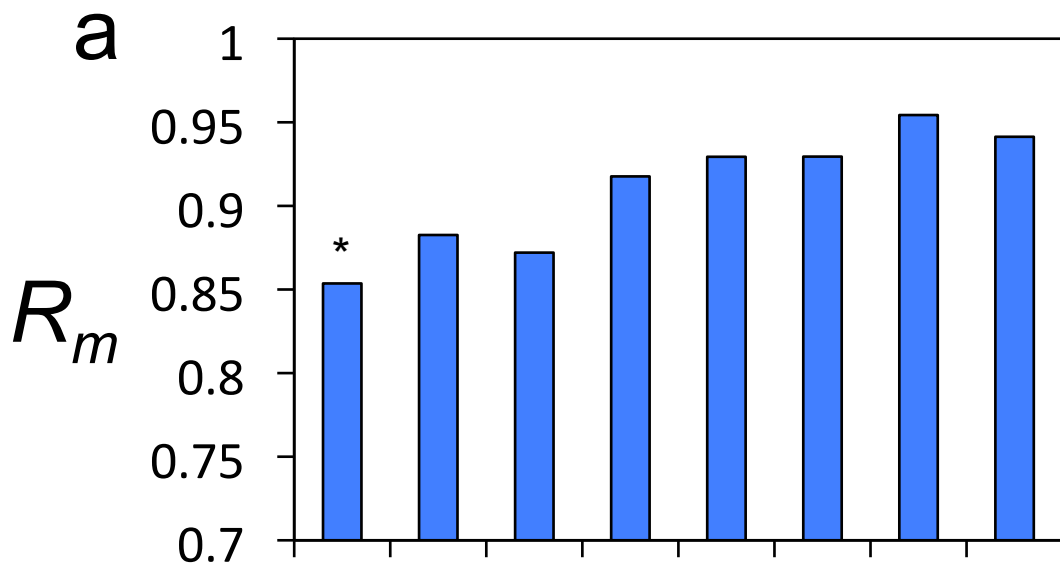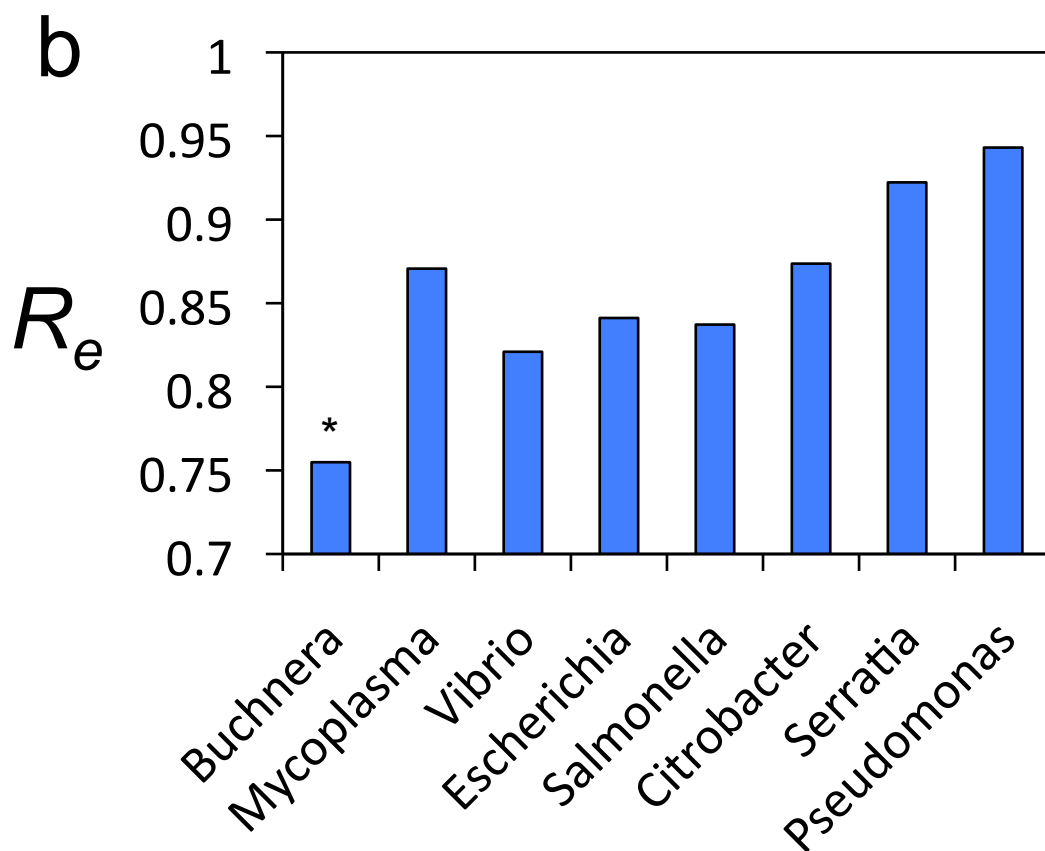

Supplement: Additional file 8 — Figure S8 Effect of environmental stability on robustness. (a) Mutational and (b) environmental robustness (Rm and Re) of gene ffs for different bacteria (Buchnera aphidicola, Mycoplasma genitalium, Vibrio fischeri, Escherichia coli, Salmonella enterica, Citrobacter koseri, Serratia proteamaculans, and Pseudomonas putida). * denotes statistical significance in a one-tailed z-test with (a) P-value = 0.059 and (b) P-value = 0.041. When including into the analysis 15 more strains of E. coli with different ffs sequences, we obtained (a) P-value = 0.005 and (b) P-value = 0.017. [file 1471-2148-12-52-S8.PDF]

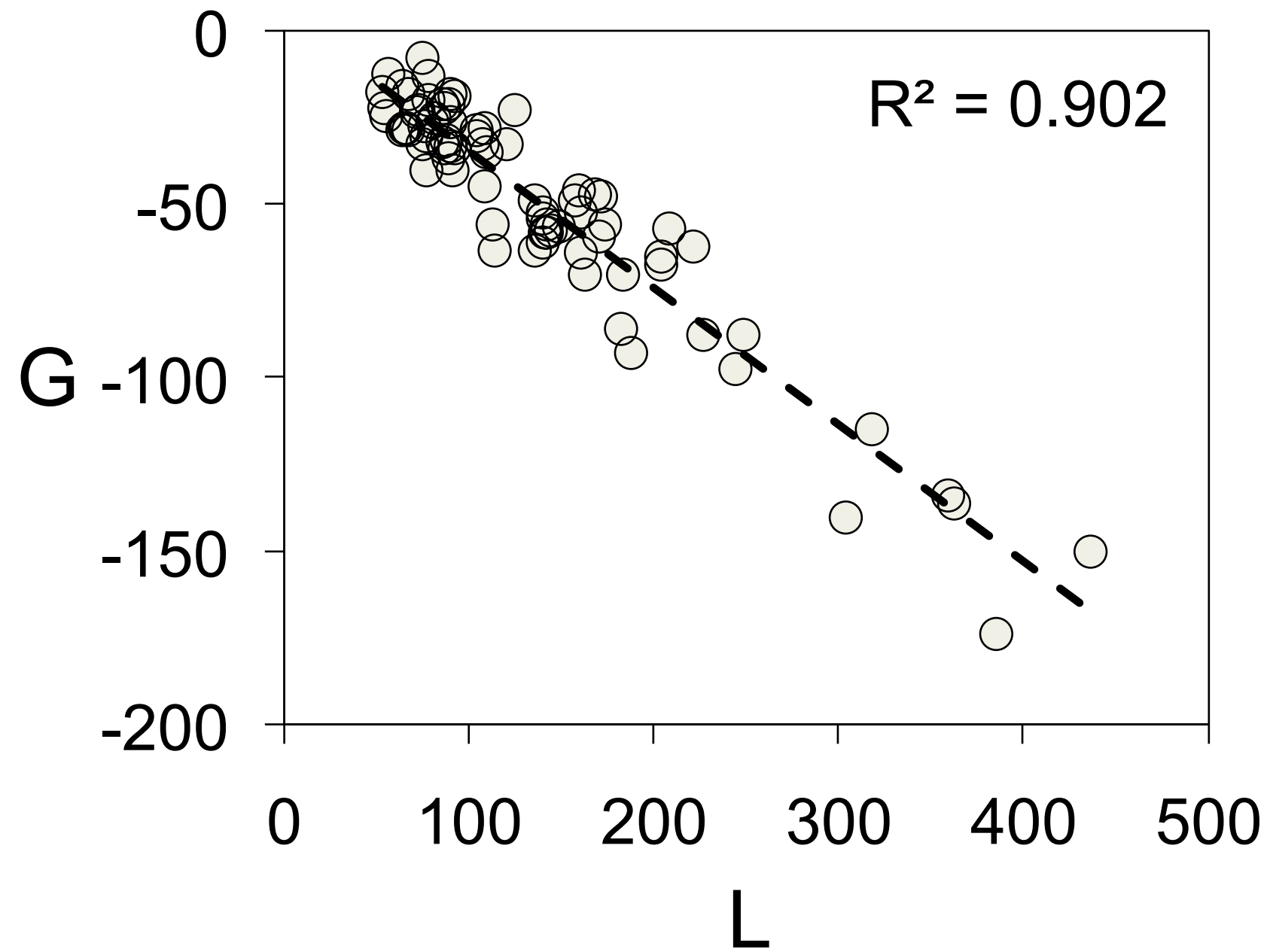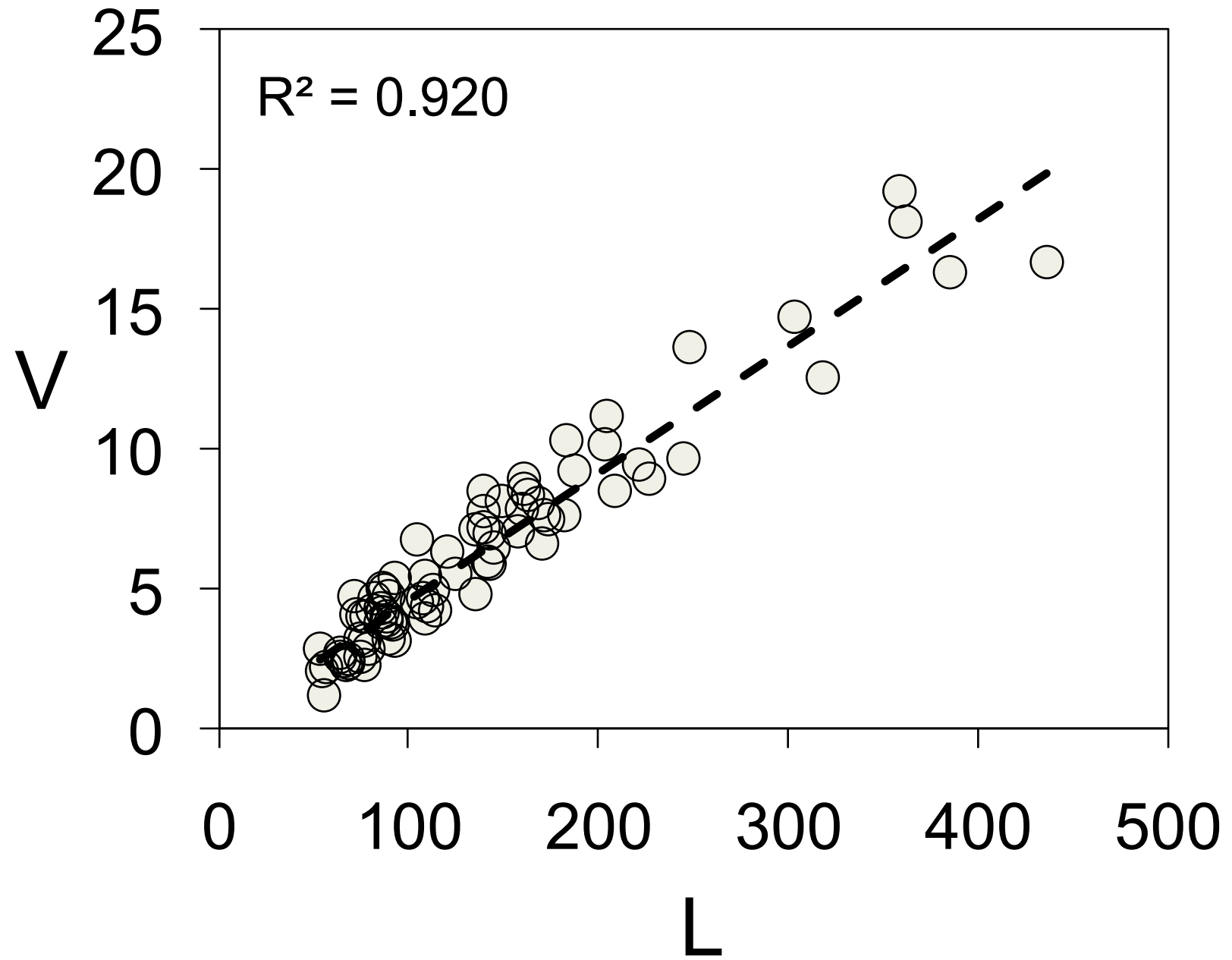

Supplement: Additional file 9 — Figure S9 Length correlates with stability and functionality. Scatter plots between length (L) and free energy of the ensemble (G) and degree of functionality (V) for the bacterial sncRNAs (G in Kcal/mol). [file 1471-2148-12-52-S9.PDF]

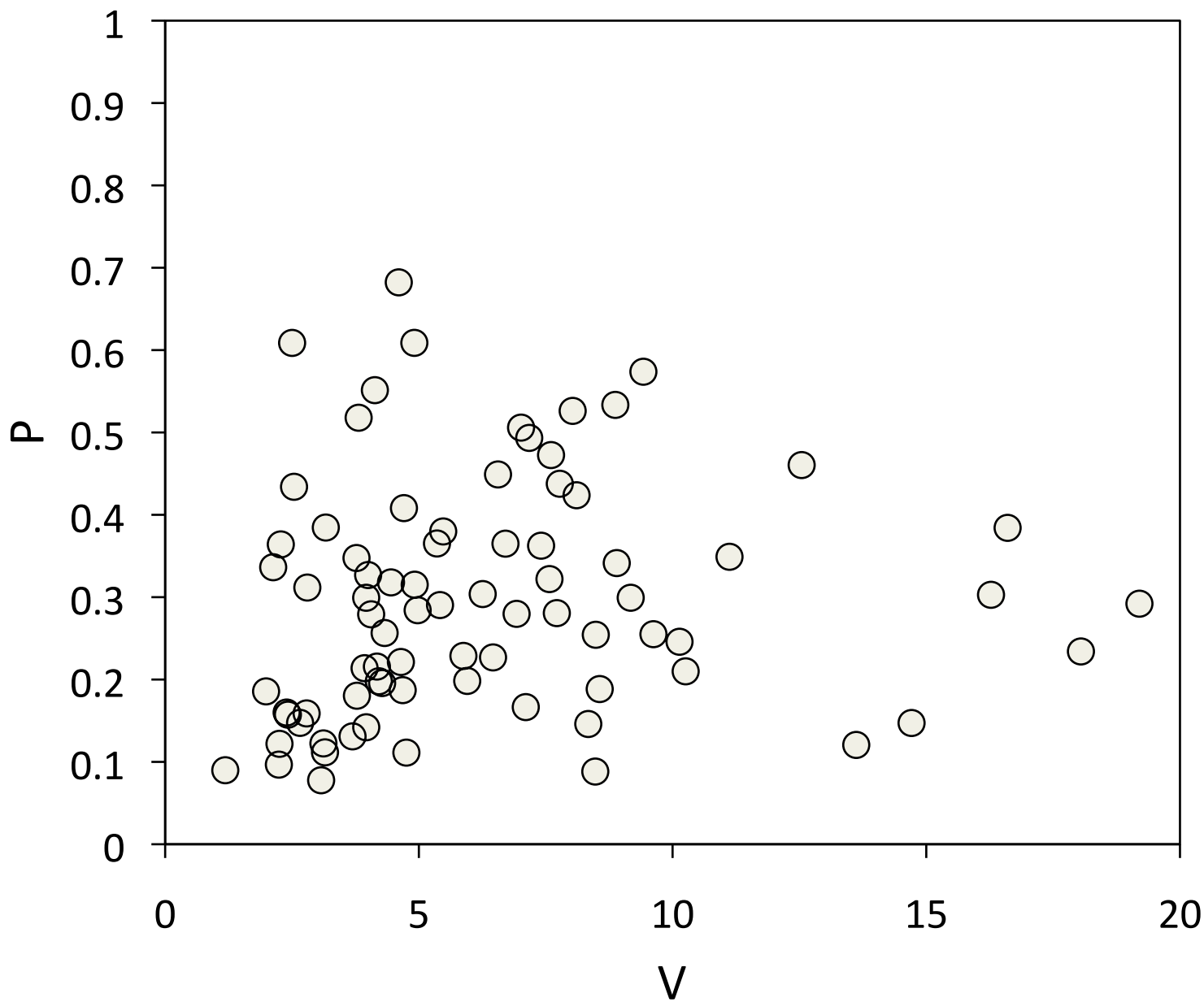

Supplement: Additional file 10 — Figure S10 Plasticity does not correlate with functionality. Scatter plot between degree of functionality (V) and plasticity (P) for the bacterial sncRNAs. [file 1471-2148-12-52-S10.PDF]
