# Supplementary material for: Describing the structural robustness landscape of bacterial small RNAs
Source: BMC Evol Biol. 2012 Apr 13;12:52. doi: 10.1186/1471-2148-12-52 (PMC3368786; doi:10.1186/1471-2148-12-52)
Supplement: Additional file 11 — Table S1 Sequences for the small non-coding RNAs (sncRNAs) obtained from the genome of the bacterium Escherichia coli K12 MG1655. [file 1471-2148-12-52-S11.PDF]

sRNA	Sequence
C0067	AUGUCUGUGGCGAAAUUGACUACCUUCGUUUUUUUGAUUAAGAAUGAUUUUAUUAUCGUAAGUAAAAUUACAUGAAUAUUUAAAAAGGAAAACGACAUGAAACCGAAGCACAGAAUCAACAUUCU
C0293	UCACCCGGGACUCGCCAGGGGACAGCCAACAGGCAUUGGGUGCAAUCACCUUAGCGUUCAGGUACAUGCGGAA
C0299	GCCACGUGAGCACAAGAUAAGAGAACGAAAAAUCAGCAGCCUAUGCAGCGACAAAUAUUGAUAGCCUGAAUCAGUAUUG
C0343	CUGUUCCAGUGCCCGAUCCGAUCCCUCGCCCGCAACCCAUGCCUGACCCACCACCCGAUGAAGAACCGAUUAAAU
C0362	AACUUUAAGCUGAAAAUGGCGCUGUAAAAGGCGCCAUUUUCAUAUUGUAGACAACGUAGGCUUUGUUCAUGCCGGAUGCGGCGUGAACGCCUUAUCCGGCAUGAAAACCCUUCAAAUCCAAUAGAUUGCAGUGAACGUGUAGGCCUGAUAAGCGUAGCGCAUCAGGCAAUGUUGCGUUUGUCAUCAGUUUCAAAUGGCGCUGUAAAAGGCGUCAUUUUCAUAUUGUAGACAACGUAGGCUUUGUUCAUGCCGGAUGCGGCGUGAACGCCUUAUCCGGCAUGAAAACCCUUCAAAUCCAAUAGAUUGCAGUGAACGUGUAGGCCUGAUAAGCGUAGCGCAUCAGGCAAUGUUGCGUUUGUCAUCAGUUCUAAAUGGCGCUUUAUAAA
C0465	GGGAAACUUUAUUGCUGAUGCCACCCGCCGCGAAAUUGAAAUAAAAAACCCGAUGCGCAGAUCAUCGGGUUCAUUUCA
C0614	GGCCGGUGUCGAUAAUACGCUAAAUAAACAAUAAUUACUCUCUUUUGCUUGACAAAAAGAGAGUUACUGGUGAGUAUUGUUUUGCUG
C0664	UGUGCCUAAAAUGUCGGAUGCGACGCUGGCGCGUCUUAUCCGACCUACGGGGACGCAUGUGUAGGCCGGAUAAGGCGUUUACGCCGCAUCCGGCAAUGGUGUCCAAAUGCAAC
C0719	UAUAGGCGUUAUACUUUACAGCAACAGUACGCCGCUAACGCAAUUGCUACCUCUGGCAUAACAAGUAUAUCGGGUAAGGGUUUCUGUUCCGCACACGCAGACGCAGAGUAUCGUUAAGAUGUCCAUAUUGUUGUUUUAGGCCCGCUAGUAAUGCGCUACGGGUAUUUAAUAUUGUUAAACCCUGAUAAUCGCUCCGGUUAUUUCCGGGAUAAAUGUACUACC
csrB	GAGUCAGACAACGAAGUGAACAUCAGGAUGAUGACACUUCUGCAGGACACACCAGGAUGGUGUUUCAGGGAAAGGCUUCUGGAUGAAGCGAAGAGGAUGACGCAGGACGCGUUAAAGGACACCUCCAGGAUGGAGAAUGAGAACCGGUCAGGAUGAUUCGGUGGGUCAGGAAGGCCAGGGACACUUCAGGAUGAAGUAUCACAUCGGGGUGGUGUGAGCAGGAAGCAAUAGUUCAGGAUGAACGAUUGGCCGCAAGGCCAGAGGAAAAGUUGUCAAGGAUGAGCAGGGAGCAACAAAAGUAGCUGGAAUGCUGCGAAACGAACCGGGAGCGCUGUGAAUACAGUGCUCCCUUUUUUUAUU
csrC	AUAGAGCGAGGACGCUAACAGGAACAAUGACUCAGGAUGAGGGUCAGGAGCGCCAGGAGGCGAAGACAGAGGAUUGUCAGGAAGACAAACGUCCGGAGACGUAAUUAAACGGAAAUGGAAUCAACACGGAUUGUUCCGGCUAAAGGAAAAACAGGGUGUGUUGGCGGCCUGCAAGGAUUGUAAGACCCGUUAAGGGUUAUGAGUCAGGAAAAAAGGCGACAGAGUAAUCUGUCGCCUUUUUUCUU
dicF	UUUCUGGUGACGUUUGGCGGUAUCAGUUUUACUCCGUGACUGCUCUGCCGCCC
dsrA	AACACAUCAGAUUUCCUGGUGUAACGAAUUUUUUAAGUGCUUCUUGCUUAAGCAAGUUUCAUCCCGACCCCCUCAGGGUCGGGAUUU
gadY	ACUGAGAGCACAAAGUUUCCCGUGCCAACAGGGAGUGUUAUAACGGUUUAUUAGUCUGGAGACGGCAGACUAUCCUCUUCCCGGUCCCCUAUGCCGGGUUUUUUU
gcvB	ACUUCCUGAGCCGGAACGAAAAGUUUUAUCGGAAUGCGUGUUCUGGUGAACUUUUGGCUUACGGUUGUGAUGUUGUGUUGUUGUGUUUGCAAUUGGUCUGCGAUUCAGACCAUGGUAGCAAAGCUACCUUUUUUCACUUCCUGUACAUUUACCCUGUCUGUCCAUAGUGAUUAAUGUAGCACCGCCUAAUUGCGGUGCUUUUUUU
IS128	UUUAGCGUAUUAUCGACACCGGCCCUUUCCGCCGUGUUCGGUAAUAAAAUAACCUGGCUUAUUAGUCCGAAUUCAGACAAAUAUAAAUAAAUCCUGCUCAAAAUUAAAAAUUCUAACCGGUAAAAGAUAUUACUUAAACAUGUAAAUUCACUUUCCUUUAAAAAACAAAAAACCGCCAAAAUCAGGCGGUUUUUUGUUGCUGGUCCGGU
isrA	UGAAAUCUGUCACUGAAGAAAAUUGGCAACUAAAGGUUAAAACCGUUAUAACACAGUCACCGGCGCAGAGGAGACAAUGCCGGAUUUAAGACGCGGAUGCACUGCUGUGUGUACUGUAGAGUCUGGCGGAUGUCGACAGACUCUAUUUUUUUAUGCAG
isrB	GACAAUAACACCUGUAUAACAAAUGGUCGGAGUGCCGCGAUGAAACUGCGCAAAAUCCUGAAAAGUAUGUUCAAUAACUAUUGCAAGACGUUCAAAGACGUACCGCCAGGCAAUAUGUUCCGAUAACAAAAAACCUGCUCCGGCAGGUUUUUUUGUGUCC
isrC	ACGAUCAAUAUCUAUUUUAUCGAUCGUUUAUAUCGAUCGAUAAGCUAAUAAUAACCUUUGUCAGUAACAUGCACAGAUACGUACAGAAAGACAUUCAGGGAACAACAGAACCACAAUUCAGAAACUCCCACAGCCGGACCUCCGGCACUGUAACCCUUUACCUGCCGGUAUCCACGUUUGUGGGUACCGGCUUUUUUAUUCACC
micA	GAAAGACGCGCAUUUGUUAUCAUCAUCCCUGAAUUCAGAGAUGAAAUUUUGGCCACUCACGAGUGGCCUUUU

micC	GUUAUAUGCCUUUAUUGUCACAGAUUUUAUUUUCUGUUGGGCCAUUGCAUUGCCACUGAUUUUCC<br>AACAUAUAAAAAGACAAGCCCGAACAGUCGUCCGGGCUUUUUUU
micF	GCUAUCAUCAUUAACUUUAUUUAUUACCGUCAUUCAUUUCUGAAUGUCUGUUUACCCCUAUUUCA<br>ACCGGAUGCCUCGCAUUCGGUUUUUUUU
omrA	CCCAGAGGUAUUGAUUGGUGAGAUUAUUCGGUACGCUCUUCGUACCCUGUCUCUUGCACCAACCU<br>GCGCGGAUGCGCAGGUUUUUUUU
omrB	CCCAGAGGUAUUGAUAGGUGAAGUCAACUUCGGGUUGAGCACAUGAAUUACACCAGCCUGCGCAG<br>AUGCGCAGGUU
oxyS	GAAACGGAGCGGCACCUCUUUUAACCCUUGAAGUCACUGCCCGUUUCGAGAGUUUCUCAACUCGA<br>AUAACUAAAGCCAACGUGAACUUUUGCGGAUCUCCAGGAUCCGCU
psrD	UAGGCAUAUUUUUUUCCAUCAGAUAUAGCGUAUUGAUGAUAGCCAUUUUAAACUAUGCGCUUCGU<br>UUUGCAGGUUGAUGUUUGUUAUCAGCACUGAACGAAAAUAAAGCAGUAACCCGCAAUGUGUGCGA<br>AUUAUUGGCAAAAGGCAACCACAGGCUGCCUUUUUCUUU
psrN	GCAAAGGGGAGUAACUUCAUUGCCGGUCGAUCGUCAUUACGAUGUGUGAAAAAACACAUCCGGUC<br>ACCGGGCAACCCGAAAGGAAUACGCAGACGUAUUCCUUUUUUGUUGUAAGUGAGACCUUGCCGGA<br>AGGCGAGGUCUAUGCAUAAAAAGCAGCGGCUGACGUCUUCCGACGUUGGCCGUUUUUU
psrO	AUCUUCUGCGCAUCCUCGCGACUAAUGACAACCCUAACCCAGCUCUAUGUGGGUAAAGCCUCUCA<br>UUAGCCGCGCGAACCUCUGCAACGGAAGAUCAUUCAUAGCAACAAUACAUUAGUUUCCAGUGAAU<br>UGCUGCCGUCAGCUUGAAAAAAGGGGCCACUCAGGCCCCCUUUU
rdlA	GUUCUGGUUCAAGAUUAGCCCCCGUUCUGUUGUCAGGUUGUACCUCUCAACGUGCGGGGGUUUUC<br>UC
rdlB	GUCUGGUUUCAAGAUUAGCCCCCGUUCUGUUGUCAGGUUUUACCUCUCAACGUGCGGGGGUUUUC<br>U
rdlC	GUCUGGUUUCAAGAUUAGCCCCCGUUUUGUUGUCAGGUUUUACCUCUCAACGUGCGGGGGUUUUC<br>UCU
rdlD	GUCUAGAGUCAAGAUUAGCCCCCGUGGUGUUGUCAGGUGCAUACCUGCAACGUGCGGGGGUUUU
rprA	ACGGUUAUAAAUCAACAUAUUGAUUUAUAAGCAUGGAAAUCCCCUGAGUGAAACAACGAAUUGCU<br>GUGUGUAGUCUUUGCCCAUCUCCCACGAUGGGCUUUUUUU
rseX	UUUUUAUUAUUCUGUGUCAUGAUGCUUCCGUUAUUAGCCUUUUAUCGUCUUGUUUAUAUUUUUUG<br>GGCCGGCAUGAUGCCGGCUUUUUUUU
rttR	CAAAAGUCCCUGAACUUCCCAACGAAUCCGCAAUUAAAUAUUCUGCCCAUGCGGGGAAGGAUGAG<br>AAGCUUCGACCAAGGUUCGACUCGAGCGCCAGCGAGAGAGCGUUGCCGCAGGCAACGACCCGAAG<br>GGCGAAGCGCGCAGCGCUGAGUAAUCCUUCCCCCACCACCA
rybA	UCAUCCCUCAAGGAUCGACGGGAUUAGCAAGUCAGGAGGUCUUAUGAAUGAGUUCAAGAGGUGUA<br>UGCGCGUGUUUAGUCAUUCUCCCU
rybB	GCCACUGCUUUUCUUUGAUGUCCCCAUUUUGUGGAGCCCAUCAACCCCGCCAUUUCGGUUCAAGG<br>UUGAUGGGUUUUUUGU
rydB	AUUAUUCUUAUCGCCCCUUCAAGAGCUAAGCCACUGAGAGUGCCGGAGAUAAGCGCCGGAUGGGG<br>UAG
rydC	CUUCCGAUGUAGACCCGUAUUCUUCGCCUGUACCACGGGUCGGUUUUAGUACAGGCGUUUUCUU
ryeA	AAAGUCAGCGAAGGAAAUGCUUCUGGCUUUUAACAGAUAAAAAGAGACCGAACACGAUUCCUGUA<br>UUCGGUCCAGGGAAAUGGCUCUUGGGAGAGAGCCGUGCGCUAAAAGUUGGCAUUAAUGCAGGCUU<br>AGUUGCCUUGCCCUUUAAGAAUAGAUGACGACGCCAGGUUUUCCAGUUUGCGUGCAAAAUGGUCA<br>AUAAAAAGCGUGGUGGUCAUCAGCUGAAAUGUUAAAAACCGCCCGUUCUGGUGA
ryeB	GCUGAUGACCACCACGCUUUUUAUUGACCAUUUUGCACGCAAACUGGAAAACCUGGCGUCGUCAU<br>CUAUUCUUAAAGGGCAAGGCAACUAAGCCUGCAUUAAUGCCAACUUUUAGCGCACG
ryeC	GUGAGGGUUAGGGAGAGGUUUCCCCCUCCCCCUGGUGUUCUUAGUAAGCCUGGAAGCUAAUCACU<br>AAGAGUAUCACCAGUAUGAUGACGUGCUUCAUCAUAACCCUUUCCUUAUUAAAAGCCCUCUUCUC<br>CGGGAGAGGCUUU
ryeD	GUGAGGGUAGAGCGGGGUUUCCCCCGCCCUGGUAGUCUUAGUAAGCGGGGAAGCUUAUGACUAAG<br>AGCACCACGAUGAUGAGUAGCUUCAUCAUGACCCUUUCCUUAUUUAUGGCCCCUUCCUCGGGAGG<br>GGCUUU
ryeE	UCGCUGAAAAACAUAACCCAUAAAAUGCUAGCUGUACCAGGAACCACCUCCUUAGCCUGUGUAAU<br>CUCCCUUACACGGGCUUAUUU
ryfA	GCGGCCCUUUCCGCCGUCUCGCAAACGGGCGCUGGCUUUAGGAAAGGAUGUUCCGUGGCCGUAAA<br>UGCAGGUGUUUCACAGCGCUUGCUAUCGCGGCAAUAUCGCCAGUGGUGCUGUCGUGAUGCGGUCU<br>UCGCAUGGACCGCACAAUGAAGAUACGGUGCUUUUGUAUCGUACUUAUUGUUUCUGGUGCGCUGU<br>UAACCGAGGUAAAUAAUAACCGGAGUCUCUCCGGCGACAAUUUACUGGUGGUUAACAACCUUCAG<br>AGCAGCAAGUAAGCCCGAAUGCCGCCCUUUGGGCGGCAUAUUUU
ryfB	CGUUAUUGAAGAUUUUGCUGUGCUUUACACCAUGCCACAGAAUUCCCCCAUUGAAACGAGUGGUG<br>UCGUCAAAGCUCUGGUGUGGAGUGCAGCAUGCACCCUCAAUAACUCGCACGUUCAGUUUUGGGGA

	GAUGUAAGGGCUAAUCUGAAUGGCUGCAUUCCUUGUUUAAGGAAAAACGAAUGACUGAUUGCCGAUACCUGAUUAAACGGGUCAUCAAAAUCAUCAUUGCUGUUUUACAGCUGAUCCUUCUGUUCUUAUAACACAAGGAAACGUACUUAAGGUGCGUCCGGUGAACCAGUCGGACGCACCUUUAAUAAC
ryfC	GUUGAGGGUGCAUGCUGCACAAAAUUAAAGUUAAAAAGUAAAACCCCCGUUCCUUACCAGUUCGGGGGUUUUACUUU
ryfD	AAUCAAGACGAUCCGGUACGCGUGAUUUUCUUUUCACAUUAAUCUGGUCAAUAACCUUGAAUAAUUGAGGGAUGACCUCAUUUAAUCUCCAGUAGCAACUUUGAUCCGUUAUGGGAGGAGUUAUGCGUCUGGAUCGUCUUACU
rygC	GUAAGGGUAAGGGAGGAUUGCUCCUCCCCUGAGACUGACUGUUAAUAAGCGCUGAAACUUAUGAGUAACAGUACAAUCAGUAUGAUGACAAGUCGCAUCAUAACCCUUCUCCUUCAAGCCCUCGCUUCGGUGAGGGCUUU
rygD	ACAAGGGUGAGGGAGGAUUUCUCCCCCCUCUGAUUGGCUGUUAAUAAGCUGCGAAACUUACGAGUAACAACACAAUCAGUAUGAUGACGAGCUUCAUCAUAACCCUUUCCUUCUGUAAGGCCCCCUUCUUCGGGAGGGGCUUUCC
rygE	ACAAGGGUAAGGGAGGAUUUCUCCCCCCUCUGAUGAGUUGUUAGUAAGUCGGGAAACUUAACAGUAACAACACAACCAGUAUGAUGACGAGCUUCAUCAUAACCCUUUCCUUAUACAAGGCCCCUUCUUCGGGAGGGGCUUU
ryhA	GUGCGGCCUGAAAAACAGUGCUGUGCCCUUGUAACUCAUCAUAAUAAUUUACGGCGCAGCCAAGAUUUCCCUGGUGUUGGCGCAGUAUUCGCGCACCCCGGUCUAGCC
ryhB	GCGAUCAGGAAGACCCUCGCGGAGAACCUGAAAGCACGACAUUGCUCACAUUGCUUCCAGUAUUACUUAGCCAGCCGGGUGCUGGCUUUU
ryjA	AUCAACACCAACCGGAACCUCCACCACGUGCUCGAAUGAGGUGUGUUGACGUCGGGGGAAACCCUCCUGUGUACCAGCGGGAUAGAGAGAAAGACAAAGACCGGAAAACAAACUAAAGCGCCCUUGUGGCGCUUUAGUUU
ryjB	UCAUCCGUCGUUGACUCCAUGCCGAUUCGGGUUAAUCUGGUAGCGAUCCCCGUCGAUACUUUUGACGAAGGCGGCAGGGAUCGCAGAAGG
sgrS	GAUGAAGCAAGGGGGUGCCCCAUGCGUCAGUUUUAUCAGCACUAUUUUACCGCGACAGCGAAGUUGUGCUGGUUGCGUUGGUUAAGCGUCCCACAACGAUUAACCAUGCUUGAAGGACUGAUGCAGUGGGAUGACCGCAAUUCUGAAAGUUGACUUGCCUGCAUCAUGUGUGACUGAGUAUUGGUGUAAAAUCACCCGCCAGCAGAUUAUACCUGCUGGUUUUUUUU
sokB	GCUAGGUUCAUUCGUUGGCCUCGGUUGAUAGAAAUAUCGGUCGGGGCCUUCGUCUU
sokC	GUUCAGCAUAUAGGAGGCCUCGGGUUGAUGGUAAAAUAUCACUCGGGGCUUUUCU
spf	GUAGGGUACAGAGGUAAGAUGUUCUAUCUUUCAGACCUUUUACUUCACGUAAUCGGAUUUGGCUGAAUAUUUUAGCCGCCCCAGUCAGUAAUGACUGGGGCGUUUUUUA
sraA	CAUUCAACGCCGAGAAUAGAGGAAAAAUUAAAGGGGAGAUAAAAUCCCCCCUUUUUG
sroA	GUUCUCAACGGGGUGCCACGCGUACGCGUGCGCUGAGAAAAUACCCGUCGAACCUGAUCCGGAUAACGCCGGCGAAGGGAUUUGAGGCUCCUU
sroB	ACACCGUCGCUUAAAGUGACGGCAUAAUAAUAAAAAAAUGAAAUUCCUCUUUGACGGGCCAAUAGCGAUAUUGGCCAUUUUU
sroC	ACUAAUUACAAGAACCAGGGGCGGAAAUUCCAGCCCUCUCGAUUGUUACGUAGCACGGACAGACUAUACGCCUGAUGGUCGUUCCCCAUCGGGCCUGAAAACCGCAAUACGCUGGGUAACAAUCUUCGAGGGUAGCAGUUAACGCUGCUACCCUCUUUUUUCU
sroD	UUACGUGACGAAGCGCGCGGCAAAGUGGACAAUAAAGCCUGAGCGUUAAGUCAGUCGUCAGACGCCGGUUAAUCCGGCGUUUUUUU
sroE	AUAACGUGAUGGGAAGCGCCUCGCUUCCCGUGUAUGAUUGAACCCGCAUGGCUCCCGAAACAUUGAGGGAAGCGUUGAGGGUUCAUUUUUAU
sroG	GCUUAUUCUCAGGGCGGGGCGAAAUUCCCCACCGGCGGUAAAUCAACUCAGUUGAAAGCCCGCGAGCGCUUUGGGUGCGAACUCAAAGGACAGCAGAUCCGGUGUAAUUCCGGGGCCGACGGUUAGAGUCCGGAUGGGAGAGAGUAACG
sroH	GAAAAUAAGAACACAUGUUCUCAUCUUCCAGGAUGCAGCAGACUGAAGAAAUUCAGACAUCCCGCAACCUGCGAUUAUCGCAAGGUCAAGGCAAAGUCCGGUAAUGGCGUUCUGAAUACCAGAGAUAAUUCUCUGGCGAAACCCACCUUAAGGUGGGUUUU
symR	AGUCAUAACUGCUAUUCUCCAGGAAUAGUGAUUGUGAUUAGCGAUGCGGGUGUGUUGGCGCACAUCCGCACCGCGCU
tff	CGGACUUCCGAUCCAUUUCGUAUACACAGACUGGACGGAAGCGACAAUCUCACUUUGUGUAACAACACACACGUAUCGGCACAUAUUCCGGGGUGCCCUUUGGGGUCGGUAAUAUGGGAUACGUGGAGGCAUAACC
tp2	ACUAAUUCUUUCGUUGCUCCAGACGACGCAGAGAACGCUCACGGCGGCUCUCUUCACGACUUCUGUCGAGCAAAAUUUCUUCGAUAAAGGCCAGAUGGCGAUGCGAUGCUUCGCGCGCUUCUUCCGGCUUACCGGCCAUAAUCGCUUCAAAUAUGCGGGUG
tpke11	UCGCCCUAUAAACGGGUAAUUAUACUGACACGGGCGAAGGGGAAUUUCCUCUCCGCCCGUGCAUU

	CAUCUAGGGGCAAUUUAAAAAAGA
tpke70	AAAGCCAUAAAAACCAUGAGGUUAUUAUGGCCGAUUUGAGGAGGGAAAGAGUAAGAGCAGUUUGU<br>UAAAUGUACAACGACGAUUCUCCCACCGGGCGCGUUUUAAAGCGACGGUGGAUCCAGAGGUACUG<br>CUCCGGUGCGCGCAUGAUCUCUUUCUCGAUAAUCUUGUUCAUAUAGGCAGCGGCUUGAUUUUCAU<br>CUGUCGGGUAGCCUUCCAUCUCUGGGGUGAUGAACAAACGAUAUCCGCUGUAAUCCGCUUUUCUU<br>ACCAUCGUUACGGUCAACAUGGCUGCGCCAGAGAGACGGGAGAGAACAUAGGUGCCAUUGGUUGU<br>GGCGACAUUUUCCACCGCAAAGAACGGCGCGAAGGAGCUGCCUUUACGACCAUAAUCCUGAUCGG<br>GAGCAAACCAUACCGCUUCACCUUUCUUCAGUGCACCGACAAUGCC
istR-1	GUUGACAUAAUACAGUGUGCUUUGCGGUUACCAGCCGCAGGCGACUGACGAAACCUCGCUCCGGC<br>GGGGUUUUUU
istR-2	GCACUAAAUACGUCAAAAUUCGUGCCGAAAUUGCGCGUUCUGCGCGGAACACGUAUACUUUCAGU<br>GUUGACAUAAUACAGUGUGCUUUGCGGUUACCAGCCGCAGGCGACUGACGAAACCUCGCUCCGGC<br>GGGGUUUUUU
ssrA	GGGGCUGAUUCUGGAUUCGACGGGAUUUGCGAAACCCAAGGUGCAUGCCGAGGGGCGGUUGGCCU<br>CGUAAAAAGCCGCAAAAAAUAGUCGCAAACGACGAAAACUACGCUUUAGCAGCUUAAUAACCUGC<br>UUAGAGCCCUCUCUCCCUAGCCUCCGCUCUUAGGACGGGGAUCAAGAGAGGUCAAACCCAAAAGA<br>GAUCGCGUGGAAGCCCUGCCUGGGGUUGAAGCGUUAAAACUUAAUCAGGCUAGUUUGUUAGUGGC<br>GUGUCCGUCCGCAGCUGGCAAGCGAAUGUAAAGACUGACUAAGCAUGUAGUACCGAGGAUGUAGG<br>AAUUUCGGACGCGGGUUCAACUCCCGCCAGCUCCACCA
ssrS	AUUUCUCUGAGAUGUUCGCAAGCGGGCCAGUCCCCUGAGCCGAUAUUUCAUACCACAAGAAUGUG<br>GCGCUCCGCGGUUGGUGAGCAUGCUCGGUCCGUCCGAGAAGCCUUAAAACUGCGACGACACAUUC<br>ACCUUGAACCAAGGGUUCAAGGGUUACAGCCUGCGGCGGCAUCUCGGAGAUUC
glmZ	GUAGAUGCUCAUUCCAUCUCUUAUGUUCGCCUUAGUGCCUCAUAAACUCCGGAAUGACGCAGAGC<br>CGUUUACGGUGCUUAUCGUCCACUGACAGAUGUCGCUUAUGCCUCAUCAGACACCAUGGACACAA<br>CGUUGAGUGAAGCACCCACUUGUUGUCAUACAGACCUGUUUU
glmY	AGUGGCUCAUUCACCGACUUAUGUCAGCCCCUUCGGGACGUGCUACAUAAAAUACGAAUGACGCA<br>CAACAAGGUGCCUGCCGUCCAACUUCUGAUAUCAGCGUAGCUAUAUCAACCAUCGGGCGAAACGU<br>CGAGUUAGGCACCGCCUUAUUCCAUAACAAAGCCGGGUAAUUCCCGGCUUUGUU
ffs	GGGGGCUCUGUUGGUUCUCCCGCAACGCUACUCUGUUUACCAGGUCAGGUCCGGAAGGAAGCAGC<br>CAAGGCAGAUGACGCGUGUGCCGGGAUGUAGCUGGCAGGGCCCCCACCC
