# Supplementary material for: Describing the structural robustness landscape of bacterial small RNAs
Source: BMC Evol Biol. 2012 Apr 13;12:52. doi: 10.1186/1471-2148-12-52 (PMC3368786; doi:10.1186/1471-2148-12-52)
Supplement: Additional file 12 — Table S2 Structural properties for the bacterial sncRNAs. These are length (L), free energy of the thermodynamic ensemble (G), degree of functionality (V), plasticity (P), mutational robustness (Rm), epistasis (E), and environmental robustness (Re). [file 1471-2148-12-52-S12.PDF]

| sRNA | L | G (Kcal/mol) | V | P | $R_m$ | E | $R_e$ |
|---|---|---|---|---|---|---|---|
| C0067 | 125 | -22.99 | 5.48 | 0.380 | 0.911 | -0.095 | 0.822 |
| C0293 | 73 | -22.97 | 4.06 | 0.279 | 0.743 | 0.134 | 0.681 |
| C0299 | 79 | -13.34 | 2.80 | 0.312 | 0.889 | -0.034 | 0.858 |
| C0343 | 75 | -7.82 | 2.54 | 0.434 | 0.864 | 0.255 | 0.847 |
| C0362 | 386 | -174.51 | 16.28 | 0.303 | 0.965 | -0.084 | 0.768 |
| C0465 | 78 | -20.26 | 3.96 | 0.299 | 0.876 | -0.052 | 0.851 |
| C0614 | 87 | -22.84 | 4.13 | 0.551 | 0.859 | 0.176 | 0.822 |
| C0664 | 113 | -56.41 | 4.91 | 0.609 | 0.755 | 0.215 | 0.577 |
| C0719 | 222 | -62.47 | 9.43 | 0.574 | 0.895 | 0.111 | 0.674 |
| csrB | 360 | -134.57 | 19.20 | 0.292 | 0.946 | 0.025 | 0.754 |
| csrC | 245 | -97.61 | 9.62 | 0.255 | 0.934 | -0.008 | 0.783 |
| dicF | 53 | -17.74 | 2.79 | 0.159 | 0.889 | -0.214 | 0.907 |
| dsrA | 87 | -33.99 | 4.98 | 0.284 | 0.909 | 0.003 | 0.873 |
| gadY | 105 | -30.71 | 6.71 | 0.365 | 0.850 | 0.123 | 0.782 |
| gcvB | 205 | -67.66 | 11.12 | 0.349 | 0.916 | 0.117 | 0.836 |
| IS128 | 209 | -57.16 | 8.49 | 0.254 | 0.961 | 0.073 | 0.883 |
| isrA | 158 | -49.23 | 7.01 | 0.506 | 0.868 | 0.164 | 0.762 |
| isrB | 160 | -46.13 | 7.78 | 0.438 | 0.922 | 0.084 | 0.831 |
| isrC | 204 | -65.24 | 10.14 | 0.246 | 0.951 | 0.128 | 0.884 |
| micA | 72 | -23.71 | 4.68 | 0.187 | 0.904 | 0.037 | 0.939 |
| micC | 109 | -28.46 | 3.93 | 0.214 | 0.908 | -0.035 | 0.866 |
| micF | 93 | -19.30 | 3.07 | 0.078 | 0.955 | -0.020 | 0.961 |
| omrA | 88 | -31.64 | 4.92 | 0.315 | 0.861 | 0.142 | 0.868 |
| omrB | 76 | -27.96 | 4.00 | 0.327 | 0.854 | 0.136 | 0.778 |
| oxyS | 110 | -35.12 | 4.33 | 0.257 | 0.915 | 0.065 | 0.867 |
| psrD | 169 | -47.37 | 8.03 | 0.526 | 0.873 | 0.144 | 0.765 |
| psrN | 188 | -93.36 | 9.18 | 0.299 | 0.940 | 0.111 | 0.879 |
| psrO | 174 | -56.26 | 7.41 | 0.363 | 0.946 | -0.033 | 0.852 |
| rdlA | 67 | -28.15 | 2.25 | 0.122 | 0.921 | 0.055 | 0.935 |
| rdlB | 66 | -28.18 | 2.39 | 0.160 | 0.928 | -0.101 | 0.920 |
| rdlC | 68 | -28.66 | 2.42 | 0.157 | 0.923 | 0.070 | 0.930 |
| rdlD | 64 | -28.76 | 2.66 | 0.147 | 0.919 | 0.002 | 0.941 |
| rprA | 105 | -29.23 | 4.45 | 0.318 | 0.887 | 0.109 | 0.892 |
| rseX | 91 | -18.77 | 3.77 | 0.348 | 0.892 | -0.134 | 0.856 |
| rttR | 171 | -59.60 | 6.56 | 0.449 | 0.886 | 0.252 | 0.794 |
| rybA | 89 | -21.15 | 3.81 | 0.518 | 0.746 | 0.227 | 0.674 |
| rybB | 81 | -26.62 | 4.17 | 0.216 | 0.908 | 0.108 | 0.894 |
| rydB | 68 | -18.25 | 2.28 | 0.364 | 0.814 | 0.287 | 0.817 |
| rydC | 64 | -15.94 | 2.50 | 0.609 | 0.705 | 0.355 | 0.762 |
| ryeA | 249 | -87.97 | 13.62 | 0.121 | 0.916 | 0.048 | 0.699 |
| ryeB | 121 | -32.85 | 6.26 | 0.304 | 0.922 | -0.112 | 0.849 |
| ryeC | 143 | -58.60 | 6.93 | 0.280 | 0.949 | 0.020 | 0.927 |
| ryeD | 136 | -63.46 | 7.11 | 0.167 | 0.944 | 0.024 | 0.895 |
| ryeE | 86 | -21.17 | 3.78 | 0.180 | 0.913 | 0.180 | 0.898 |
| ryfA | 304 | -140.46 | 14.71 | 0.147 | 0.942 | -0.054 | 0.731 |
| ryfB | 319 | -115.30 | 12.54 | 0.460 | 0.913 | 0.148 | 0.724 |
| ryfC | 77 | -30.71 | 3.12 | 0.122 | 0.926 | 0.152 | 0.950 |
| ryfD | 143 | -56.43 | 5.88 | 0.229 | 0.897 | 0.040 | 0.741 |
| rygC | 140 | -61.63 | 8.48 | 0.088 | 0.933 | -0.227 | 0.875 |
| rygD | 145 | -57.88 | 6.46 | 0.227 | 0.940 | -0.115 | 0.839 |
| rygE | 142 | -58.40 | 5.95 | 0.198 | 0.918 | 0.103 | 0.865 |

| | | | | | | | |
|---|---|---|---|---|---|---|---|
| ryhA | 108 | -32.75 | 4.60 | 0.682 | 0.846 | 0.117 | 0.714 |
| ryhB | 90 | -26.39 | 4.71 | 0.408 | 0.824 | 0.260 | 0.812 |
| ryjA | 140 | -54.40 | 7.72 | 0.281 | 0.929 | 0.146 | 0.842 |
| ryjB | 90 | -33.58 | 3.17 | 0.385 | 0.757 | 0.227 | 0.785 |
| sgrS | 227 | -87.79 | 8.90 | 0.341 | 0.919 | 0.126 | 0.793 |
| sokB | 56 | -24.94 | 1.19 | 0.090 | 0.901 | 0.124 | 0.963 |
| sokC | 55 | -22.45 | 1.99 | 0.186 | 0.921 | 0.088 | 0.956 |
| spf | 109 | -45.29 | 5.42 | 0.290 | 0.856 | 0.191 | 0.831 |
| sraA | 57 | -12.89 | 2.13 | 0.336 | 0.896 | 0.090 | 0.869 |
| sroA | 93 | -33.88 | 5.36 | 0.365 | 0.851 | 0.173 | 0.814 |
| sroB | 82 | -25.30 | 4.65 | 0.221 | 0.932 | -0.089 | 0.917 |
| sroC | 163 | -70.61 | 8.34 | 0.146 | 0.954 | -0.095 | 0.908 |
| sroD | 86 | -32.47 | 4.28 | 0.196 | 0.889 | 0.177 | 0.871 |
| sroE | 92 | -40.66 | 3.69 | 0.131 | 0.934 | 0.041 | 0.942 |
| sroG | 149 | -56.90 | 8.11 | 0.424 | 0.860 | 0.189 | 0.710 |
| sroH | 161 | -64.20 | 8.56 | 0.188 | 0.934 | 0.010 | 0.882 |
| symR | 77 | -40.65 | 2.24 | 0.097 | 0.941 | -0.124 | 0.969 |
| tff | 136 | -49.32 | 4.75 | 0.111 | 0.943 | -0.188 | 0.869 |
| tp2 | 161 | -52.89 | 8.87 | 0.533 | 0.893 | 0.065 | 0.714 |
| tpke11 | 89 | -37.09 | 3.96 | 0.142 | 0.952 | -0.088 | 0.929 |
| tpke70 | 436 | -150.42 | 16.60 | 0.384 | 0.940 | -0.095 | 0.609 |
| istR-1 | 75 | -33.22 | 3.15 | 0.112 | 0.899 | 0.181 | 0.908 |
| istR-2 | 140 | -52.87 | 7.18 | 0.493 | 0.889 | 0.058 | 0.739 |
| ssrA | 363 | -136.43 | 18.05 | 0.234 | 0.925 | -0.147 | 0.529 |
| ssrS | 183 | -86.10 | 7.57 | 0.322 | 0.919 | 0.105 | 0.819 |
| glmZ | 172 | -47.86 | 7.61 | 0.473 | 0.903 | 0.119 | 0.780 |
| glmY | 184 | -70.41 | 10.26 | 0.210 | 0.931 | -0.050 | 0.795 |
| ffs | 114 | -63.39 | 4.21 | 0.198 | 0.918 | 0.011 | 0.841 |
