# Supplementary material for: Describing the structural robustness landscape of bacterial small RNAs
Source: BMC Evol Biol. 2012 Apr 13;12:52. doi: 10.1186/1471-2148-12-52 (PMC3368786; doi:10.1186/1471-2148-12-52)
Supplement: Additional file 13 — Table S3 Statistical significance analysis results. Z-scores for plasticity (P), mutational robustness (Rm), environmental robustness (Re), and epistasis (E), for each sncRNA and relative to sample III. [file 1471-2148-12-52-S13.PDF]

RNA_Identifier	Z_P	Z_Rm	Z_Re	Z_E
C0067	1.49562746	-0.0061504	-0.4605997	-1.1020749
C0293	-0.5566721	-1.3279765	-1.2019498	0.15453856
C0299	-0.3134244	0.63911404	0.51518727	-0.7867676
C0343	-0.1495156	0.88161639	1.71069756	0.77146927
C0362	1.2644488	0.04967218	-1.7803012	-1.4018757
C0465	0.30595593	0.7072189	0.44352692	-0.8567731
C0614	4.56035388	-0.5225252	-1.1142026	1.05290751
C0664	7.32530397	-5.8709215	-5.8242166	1.96380425
C0719	3.7832488	-1.4467857	-2.0257832	1.13655189
csrB	-0.4720944	0.56281314	0.50621973	-0.3685212
csrC	-0.0856061	-0.0893067	-0.3907217	-0.4151429
dicF	-0.1681373	0.85338162	0.58823222	-1.1405404
dsrA	6.22545188	-1.4165497	-3.1306208	0.34570299
gadY	-0.0030538	-0.1477389	0.23225577	0.12582051
gcvB	0.69224038	0.02621865	1.04750205	0.70605294
IS128	-0.6163932	1.56453709	1.7319654	0.04176026
isrA	1.46487311	-1.6687354	-0.9055655	0.8098095
isrB	0.95242765	0.60270168	0.70181122	0.38729079
isrC	-0.8294483	1.30084105	1.61436568	0.67457459
micA	-0.1462394	1.06583587	1.53915548	-0.1141667
micC	-0.655602	0.30915184	0.42271116	-0.5610251
micF	-2.9225684	2.05170847	2.34447667	-0.5398409
omrA	0.93368053	-0.6620052	0.12989379	0.81213774
omrB	0.85434103	0.01351042	-0.8169768	0.52526283
oxyS	-0.2704312	1.28111309	1.16883259	-0.2058857
psrD	2.64299099	-0.6890685	-0.2271475	0.90217345
psrN	1.83287247	0.20257562	0.21890088	0.68899952
psrO	1.01929361	1.13094081	1.00975107	-0.4048911
rdlA	-0.1320498	0.53966861	0.41920544	0.55495108
rdlB	0.47955028	0.7273934	0.14092059	-0.2593911
rdlC	0.29111774	0.53336741	0.46525874	0.61159335
rdlD	0.79264914	0.60439984	0.42372816	0.19593295
rprA	-0.112185	0.13829964	1.24304577	0.23636347
rseX	0.80047307	0.25508327	0.23143158	-1.530634
rttR	2.11304058	-0.6772697	-0.3141168	1.64042772
rybA	1.24439164	-1.0763696	-0.9589321	0.71447624
rybB	-0.2941661	0.21303385	0.37058853	0.75324835
rydB	-0.2257008	-0.3238714	0.52053836	0.9938271
rydC	2.59008535	-2.0446529	-1.300292	1.49112798
ryeA	-1.6301484	-0.8974802	-1.5155736	0.19299269
ryeB	-0.4436299	1.31606206	1.62869746	-1.56546
ryeC	1.58212648	0.86468129	0.9732936	0.2506693
ryeD	0.07226749	0.92717831	0.5285771	0.3196852
ryeE	-1.0024684	0.65507538	0.87008131	0.88910092
ryfA	-0.5715238	-0.6452783	-1.9151809	-0.5654575
ryfB	3.51416253	-1.6595047	-1.318357	1.45869072
ryfC	-0.3707025	0.60948439	1.08685576	1.20691057
ryfD	0.43893955	-0.6942178	-1.8032955	0.11482148
rygC	-0.7173821	0.40264158	0.06439387	-1.249181
rygD	0.80430781	0.52947043	-0.2273423	-0.455914
rygE	-0.291532	0.85551376	0.91772193	0.43456077
ryhA	1.9029886	-0.2729423	-1.0510732	0.24782753
ryhB	0.11627278	-0.0337973	0.78537775	1.16729683
ryjA	-0.3654838	1.26294003	0.9398798	0.62085617
ryjB	1.65206256	-2.2091133	-0.8791149	1.31132274
sgrS	1.17297564	-0.9334598	-0.7713318	1.15650828
sokB	-0.7800908	0.56125694	0.96920883	0.81514528
sokC	2.78563916	0.5977091	-0.3051971	0.54205567
spf	1.13182005	-1.0780235	-0.4164454	1.29918237
sraA	0.91068803	1.20766039	0.70489988	0.04932692
sroA	0.04126355	-0.0099946	0.61060744	0.57009591
sroB	-0.0850017	1.30093311	0.87954481	-1.2208726
sroC	-1.6608659	1.25650147	1.29629379	-1.0022332
sroD	-0.4124373	0.41203417	0.35286861	0.76290222
sroE	-0.5569606	0.89561537	1.19623401	0.13360977
sroG	1.13448505	-0.9200151	-1.415216	1.21257741
sroH	-0.5247294	0.70097728	0.99346966	0.02865103
symR	1.45143709	1.35794629	0.05387165	-1.4320135
tff	-1.1555481	0.82574381	0.53156964	-1.2976218
tp2	1.38463361	0.11223039	-0.5078375	-0.0918672
tpke11	-0.6710521	1.63007721	1.39736044	-0.3853822
tpke70	0.89121676	-0.1756376	-1.4208311	-1.1918868
istR-1	-0.0760238	-0.0997715	-0.1321753	1.30895537
istR-2	2.81724697	-0.4788784	-1.3079856	0.44762482
ssrA	-0.5560971	-0.4240352	-2.5206777	-1.6139635
ssrS	1.45641569	-0.4940807	-0.5874147	0.85592067
glmZ	0.97119173	0.66912212	0.77258384	0.19364778
glmY	-0.5222321	0.58724699	0.16764345	-0.5283533
ffs	0.47069996	0.06294372	-0.3211952	0.28694875
