# Supplementary material for: Describing the structural robustness landscape of bacterial small RNAs
Source: BMC Evol Biol. 2012 Apr 13;12:52. doi: 10.1186/1471-2148-12-52 (PMC3368786; doi:10.1186/1471-2148-12-52)
Supplement: Additional file 14 — Table S4 Effect of random sample of sequences on robustness. Sample I accounts for sequences obtained from inverse folding routines. Sequences of sample II were subsequently randomized by introducing neutral mutations (which not change the structure). Sequences of sample III were randomized by introducing neutral mutations and neutral pairs of mutations and compensatory mutations (for nucleotides in a stem). Using z-scores, we show the percentage of sncRNAs that are robust (z > 0) and significantly robust (zc = 1.64, P-value = 0.05). [file 1471-2148-12-52-S14.PDF]

	Sample I		Sample II		Sample III	
	$z>0$	$z>z_c$	$z>0$	$z>z_c$	$z>0$	$z>z_c$
R_m	91.1	31.6	84.8	22.8	60.8	1.3
R_e	91.1	32.9	84.8	26.6	59.5	3.8
